# Mechanotransductive cascade of Myo-II-dependent mesoderm and endoderm invaginations in embryo gastrulation

**DOI:** 10.1038/ncomms13883

**Published:** 2017-01-23

**Authors:** Démosthène Mitrossilis, Jens-Christian Röper, Damien Le Roy, Benjamin Driquez, Aude Michel, Christine Ménager, Gorky Shaw, Simon Le Denmat, Laurent Ranno, Frédéric Dumas-Bouchiat, Nora M. Dempsey, Emmanuel Farge

**Affiliations:** 1 Mechanics and Genetics of Embryonic Development group, Institut Curie, PSL Research University, CNRS, UMR168, Inserm, Marie Curie Univ Paris 06, Institut Curie, 11 rue Pierre et Marie Curie, 75005 Paris, France; 2 Univ. Grenoble Alpes, Inst NEEL, F-38042 Grenoble, France; 3 CNRS, Inst NEEL, F-38042 Grenoble, France; 4 Sorbonne Universités, Université Pierre et Marie Curie Univ Paris 06, Laboratoire PHENIX-UMR 8234, 1 Place Jussieu, 75005 Paris, France

## Abstract

Animal development consists of a cascade of tissue differentiation and shape change. Associated mechanical signals regulate tissue differentiation. Here we demonstrate that endogenous mechanical cues also trigger biochemical pathways, generating the active morphogenetic movements shaping animal development through a mechanotransductive cascade of Myo-II medio-apical stabilization. To mimic physiological tissue deformation with a cell scale resolution, liposomes containing magnetic nanoparticles are injected into embryonic epithelia and submitted to time-variable forces generated by a linear array of micrometric soft magnets. Periodic magnetically induced deformations quantitatively phenocopy the soft mechanical endogenous *snail*-dependent apex pulsations, rescue the medio-apical accumulation of Rok, Myo-II and subsequent mesoderm invagination lacking in *sna* mutants, in a Fog-dependent mechanotransductive process. Mesoderm invagination then activates Myo-II apical accumulation, in a similar Fog-dependent mechanotransductive process, which in turn initiates endoderm invagination. This reveals the existence of a highly dynamic self-inductive cascade of mesoderm and endoderm invaginations, regulated by mechano-induced medio-apical stabilization of Myo-II.

## Abstract (web summary)

Mechanical signals regulate tissue differentiation but how this triggers downstream biochemical signals is unclear. Here, the authors place micro-magnets in the *Drosophila* embryonic epithelia and show this triggers apical pulsations, in turn stabilizing Myosin-II, resulting in mesoderm invagination.

Both biochemical patterning and biomechanical morphogenesis contribute to shaping living organisms during embryonic development. The role of biochemical and genetic information in the development of a functional three-dimensional tissue morphology is beginning to be well understood in different developmental contexts^1^,^2^,^3^,^4^,^5^,^6^,^7^,^8^,^9^. The reverse role of mechanical cues developed by biomechanical morphogenetic movements, in the activation of developmental biochemical pathways involved in the triggering of cell differentiation, is emerging as a key feature of embryonic development regulation^10^,^11^,^12^. Mechanical cues generated by developmental strain deformation were found to activate major developmental differentiation events, including early anterior endoderm and mesoderm specification^13^,^14^, antero-posterior axis formation^15^, tooth formation^16^ and joint bone differentiation^17^.

Mechanical signalling has also been suggested to trigger the active morphogenetic movements, including mesoderm invagination^18^, folding in legs and wings during organogenesis^19^, primitive streak formation in chick embryos^8^, and cell–cell junction re-enforcement^20^,^21^ in animal development, as well as meristem morphogenesis and increased growth rate in plant development^22^,^23^. Strains developed by one morphogenetic movement were also suggested to mechanically influence another, for example, with posterior endoderm invagination promoting vertex resolution during germ-band extension^24^,^25^. However, the tools used so far to test mechanical triggering of active morphogenetic movements in the context of morphogenetic deficiencies were based on the use of either soft but non-physiological direct mechanical deformation (such as indentation or pipette aspiration) or of genetically induced physiological deformation, which is not directly mechanical (Myosin-II (Myo-II) activation in neighbouring cells). This opened the possibility of a response to non-physiological mechanical strains or to uncontrolled indirect biochemical pathways independent of mechanical strains, for instance of a Myo-II-triggered re-enforcement of cell–cell junctions potentially leading to Myo-II recruitment in neighbouring cells in a purely biochemical process, respectively.

By developing an innovative micro-magnet-based tool capable of producing dynamic physiological direct mechanical strain *in vivo* with cell scale resolution, here we show mechanical triggering of active morphogenetic movements by the mechanical cues induced by preceding morphogenetic movements. Specifically, apical constriction of mesodermal cells, and the resulting mesoderm invagination, which corresponds to the first morphogenetic movement of early *Drosophila* embryogenesis, are both demonstrated to be mechanically activated by soft apical pulses of the mesoderm.

In the *Drosophila* embryo, mesoderm invagination represents the first morphogenetic movement of embryogenesis. This movement is triggered by medio-apical stabilization of Myo-II, leading to the acto-myosin apical constriction of mesoderm apexes that generates tissue bending and folding^26^,^27^. Apical constriction follows two distinct phases. The first phase is characterized by *snail* (*sna*)-dependent uncoordinated unstable constricting pulsations, associated with unstable medio-apical coalescence of Myo-II spots. The second phase, which leads to invagination, is characterized by a *twist* (*twi*)-dependent coordinated stable constriction, associated with stable and progressive coalescence of medio-apical Myo-II spots^26^,^28^ (Fig. 1a). In *sna* mutants, both the uncoordinated and coordinated phases are absent, as is mesoderm invagination, thus revealing the interplay between *sna* and *twi* pathways in the regulation of mesoderm invagination^26^,^28^. Medio-apical stabilization of Myo-II, collective apical constriction and mesoderm invagination can be mechanically rescued in *sna* mutants by indenting the mesoderm by a few microns. This stimulates a mechanosensitive pathway, Folded gastrulation (Fog), that is downstream of *twist*^18^. Such a response to a non-physiological mechanical perturbation suggested that the mechanotransductive mechanical induction of medio-apical stabilization of Myo-II by the physiological mechanical strains resulting from *sna*-dependent pulsations could regulate the interplay between *sna* and *twi* pathways, which in turn leads to mesoderm morphogenesis.

We show that the medio-apical stabilization of Myo-II in early *Drosophila* embryo mesoderm is mechanotransductively triggered in response to strain developed by *sna*-dependent pre-existing individual cell apex pulsations. This is demonstrated by mimicking the physiological apical pulsations absent in *sna* mutants, resulting in the rescue the Fog-dependent medio-apical stabilization of Rho*-*associated protein kinase (Rok) and Myo-II, and then mesoderm invagination. Pulsations are induced using time varying, cell scale spatially localized mechanical forces generated within *sna* mutant embryos, by exploiting magnetic interactions between magnetic nanoparticles (MNPs) encapsulated into liposomes loaded within the embryo, and a near-by array of micro-magnets. In addition, we find that mesoderm constriction and folding leads to the stretching of posterior endoderm cells, which in turn activates the apical accumulation of Myo-II required for its invagination, in a Fog-dependent mechanotransductive process.

Embryonic development has long been considered to be the product of an inductive cascade of developmental gene expression^29^,^30^. Our results reveal the additional existence of a rapid self-inducting mechanotransductive cascade of active morphogenetic events, driving the sequence of apical stabilization of Myo-II of the first two invaginations in the morphogenesis of early *Drosophila* embryos. The cascade consists of the *sna*-dependent apex pulsations triggering medio-apical stabilization of Myo-II and mesoderm invagination, which in turn mechanically activates the apical stabilization of Myo-II that triggers posterior endoderm invagination.

## Results

### Micro-magnets to mimic *sna*-dependent mechanical pulsations

**Figure 1:**
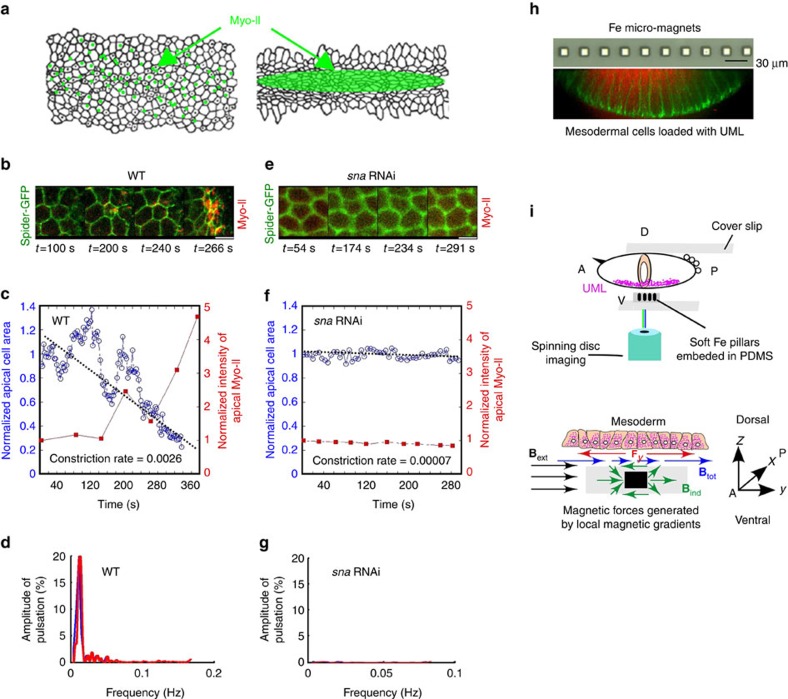
Use of magnetic forces to mimic *sna*-dependent apex pulsations in mesoderm cells. (**a**) Snail-dependent stochastic fluctuation of mesoderm apex size followed by the medio-apical accumulation of Myo-II. (**b**) Apex size pulsations imaged in the time range 100–240 s, followed by cell constriction (image at 266 s) monitored using Spider–GFP (in green), with submembrane apical accumulation of Myo-II (sqh–mCherry in red). (**c**) Variation of the apical surface area (in blue) and Myo-II concentration (in red) as a function of time in the WT. Representative of the *n*=10 quantified on the *N*=64 pulsating cells, of the 587 mesoderm cells observed, in six distinct embryos. (**d**) Spectral signature of *sna*-dependent pulsations in the WT before stable constriction, following Fourier transformation of **c**. Representative of the *n*=8 cells allowing analysis (that is, staying in the same focus plane long enough) on the *N*=64 pulsating cells of the 587 mesoderm cells observed in six distinct embryos. (**e**) Absence of cell pulsations, constriction and submembrane apical accumulation of Myo-II, in *sna* RNAi (*n*=6 on *N*=6 embryos). (**f**) Variation of the apical surface area (in blue) and Myo-II concentration (in red) as a function of time in *sna* RNAi. Representative of the *n*=17 quantified cells of the 892 mesoderm cells observed in six distinct embryos. (**g**) Spectral signature of *sna* RNAi, following Fourier transformation of **f**. (**h**) Up: optical microscope image (reflection mode) of the micro-magnet network embedded in PDMS. Down: image of fluorescent UML (red) inside the basal domain of mesoderm cells, following the completion of cell formation at the end of stage 5. Green is Spider–GFP. (**i**) Up: schematic of the confocal-spinning disk microscope set-up in which the UML-loaded embryo is positioned on top of a transparent PDMS membrane containing a linear array of micro-magnets. Down: schematic of the homogeneous external magnetic field, **B**_ext_ (in black) used to magnetize the soft micro-magnets, producing an inhomogeneous induced field, **B**_ind_ (in green). The gradient of the induced field creates an in-plane tangential repulsive force *F_y_* (in red) on UML, magnetized by **B**_tot_ (in blue). A is anterior and P is posterior. White scale bars are 5 μm.

**Figure 2:**
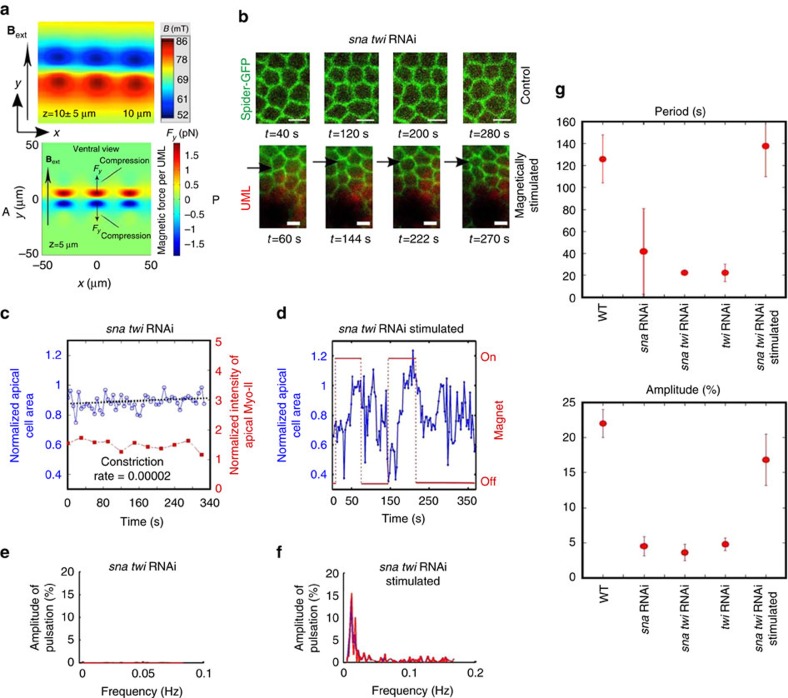
Mimicking of *sna*-dependent apex pulsations in mesoderm cells. (**a**) Up: ventral view of the magnetic field measured on the apical surface of mesoderm cells. Quantitative estimate of the stray field produced in the vicinity of the micro-magnets, measured by scanning hall probe microscopy (probe tilted by 23°) at a distance of 10±5 μm above the micro-magnets. Bottom: ventral view of the magnetic force *F*_*y*_ on the apex of the mesoderm cells. Simulation of the in-plane (*y* component) magnetic force experienced by an UML positioned in the plane 5 μm above the micro-magnets. (**b**) Magnetic stimulation of apex pulsations by the application of a pulsed magnetic field to micro-magnets (shadowy object at bottom of images) positioned close to UML-loaded mesoderm cells on *sna twi* spider–GFP embryos. Green corresponds to Gilgamesh and red to UML. (**c**) Variation of the apical surface (in blue) and Myo-II concentration (in red) as a function of time in the *sna twi* RNAi. Representative of the *n*=11 quantified cells of the *N*=745 cells observed in six distinct embryos. (**d**) Variation of the apical surface area (in blue) of cell pulsations induced by the application of a pulsed magnetic field to micro-magnets positioned close to UML-loaded mesoderm cells, in *sna twi* RNAi. Representative of the *n*=12 quantified cells of the 71 pulsating cells on the *N*=726 cells observed in six distinct embryos. (**e**) Spectral signature of apex surface area fluctuations in the mesoderm cells of *sna twi* RNAi embryos. Representative of the *n*=3 quantified cells of the *N*=745 cells observed in six distinct embryos. (**f**) Spectral signature of magnetically induced apex surface pulsations in the mesoderm cells of *sna twi* RNAi embryos. Representative of the *n*=12 quantified cells of the 55 pulsating cells on the *N*=596 cells observed in five distinct embryos. (**g**) Period and amplitude of mesoderm cell apex pulsations in the WT, versus *sna* RNAi (*P*_per_=0.006, *P*_amp_=0.002), *sna twi* RNAi (*P*_per_=0.01, *P*_amp_=0.01)*, twi* RNAi (*P*_per_=0.007, *P*_amp_=0.002), consistently repressing the pulsating effects of its *sna* target gene in the presumable case *twi* RNAi is efficient enough, and *sna twi* RNAi magnetically stimulated (*P*_per_=0.4, *P*_amp_=0.005), and *sna twi* RNAi versus *sna twi* RNAi magnetically stimulated embryos (*P*_per_=0.013, *P*_amp_=0.011). *P* values are calculated from the Mann–Whitney test, and error bars are s.d.

During the early stages of *Drosophila* embryo development, *sna*-dependent mesoderm cell apex pulsations initiate at the end of mesoderm cell formation^31^. Using the GFP-tagged transmembrane protein Gilgamesh (Spider–GFP), that labels cell plasma membranes, and Myo-II, which labels the light chain of Myo-II (Myo-II–mCherry), we monitored the change in the surface area of the apex of mesoderm cells and the accumulation of apical Myo-II, respectively^28^. The pulsating phase associated with pulsatile unstable submembranar accumulation of Myo-II^28^, initiated 2 min before the end of cellularization, which was defined as time 0 for all experiments. This phase was observed to last 3 min (Fig. 1b,c). We subsequently observed the initiation of the stable constricting phase, associated with medio-apical stabilization of Myo-II, which drives mesoderm invagination (Fig. 1b,c)^28^. Fourier transform analysis of the pulsating phase showed a spectral signature characterized by a mean amplitude of relative surface area 22±2% with a mean period of 126±22 s (Figs 1d and 2g). In *sna* RNA interference (RNAi) injected Spider–GFP Sqh–mCherry embryos, both the pulsating and stably constricting phases are absent, no characteristic period emerges from the spectral signature and there was no apical accumulation of Myo-II (Figs 1e–g and 2g)^28^. The pulsatile phase was consistently suppressed after injection of the Y27632 inhibitor of Rok that is required for Myo-II activity (Supplementary Fig. 1a)^32^,^33^.

To test for the triggering of medio-apical stabilization of Myo-II by the mechanical strain generated on mesoderm cells during *sna*-dependent pulsations, we developed an innovative approach involving the use of internalized MNPs encapsulated into liposomes and an external array of soft micro-magnets. The soft micro-magnets are magnetized by an external macroscopic magnetic field produced by an electromagnet operated in pulsed mode. When magnetized, the soft micro-magnets produce spatially varying high magnetic field gradients, which serve to apply pulsed forces on superparamagnetic nanoparticles internalized in mesoderm cells (Fig. 1h,i; Supplementary Figs 1b and 2a). By magnetizing the micro-magnets in a pulsed manner, we effectively apply a pulsed force. The resulting dilating-constricting mechanical forces produced within the cells neighbouring the magnets were used for *in vivo* quantitative mimicking of apex pulsations.

Specifically, the design of the micro-magnets was guided by the fact that in the wild type (WT), both pulsating cells and first constricting cells are found along the ventral midline of the mesoderm. A linear array of iron micro-magnets, approximately cubic in shape and of edge length 10 μm, separated by a distance of 20 μm, was embedded just below the surface of a transparent polydimethylsiloxane (PDMS) membrane of total thickness close to 50 μm (Fig. 1i; Supplementary Fig. 2a,b; for fabrication details see the Methods section). Liposomes containing superparamagnetic particles, hereafter referred to as ultra-magnetic liposomes (UMLs, see the Methods section)^34^, were injected into the ventral cells of embryos (Fig. 1h and Supplementary Fig. 1b). Once injected embryos were positioned ventrally at a distance of 5±2 μm from the micro-magnets (Fig. 1i), a macroscopic homogenous external magnetic field of 180 mT, produced by an electromagnet operated in pulsed ‘60 s on–60 s off' mode, was applied in the plane of the micro-magnet array, perpendicular to the linear axis of the array (Supplementary Fig. 2b).

The superparamagnetic particles are subjected to forces induced by the total magnetic field gradient ∇ (*B*_tot_) on the length scale of the micro-magnet size, that is, of the order of tens of microns (Fig. 1i; Supplementary Fig. 2a–d). These forces are proportional to and in the direction of the gradient of the magnetic field amplitude (that is, of the strongest magnetic field *B*_tot_ amplitude: **F***=m*_sat_ ∇ (*B*_tot_))^35^, if magnetic moments *m*_sat_ are saturated by the magnetic field, which, as a good approximation, is the case everywhere at *z*=5±2 μm from the surface of the magnets (Fig. 1i; see Methods).

The external magnetic field produced by the electromagnet **B**_ext_ serves to magnetize the soft iron micro-magnets, thus generating a magnetic dipole hereafter referred to as the induced field **B**_ind_ around each micro-magnet, which leads to the local magnetic field gradients (Fig. 1i; Supplementary Fig. 2c). The existence of this induced local magnetic field gradient was experimentally demonstrated with a scanning hall probe microscope (SHPM), the mean field profile being in quantitative agreement with simulations (Fig. 2a, up; Supplementary Fig. 2d; Methods).

The induced magnetic field **B**_ind_ above and below the micro-magnets opposes to the external magnetic field **B**_ext_, and leads to a reduced total magnetic field amplitude of **B**_tot_=**B**_ext_+**B**_ind_ compared with the amplitude **B**_ext_ (Fig. 1i; Supplementary Fig. 2c). Therefore, the presence of the pillar generates local gradients of the total magnetic field amplitude **B**_tot_ with smaller values directly above and below the pillar compared with above and below the edges of the pillar (Fig. 1i; Supplementary Fig. 2c, down). The magnetic forces applied to superparamagnetic UML-loaded cells being proportional to, and aligned along the magnetic field gradient, this generates magnetic forces applied on the cells along *y*, orientated from above the pillar to away from the pillar (Fig. 1i). The existence of such repulsive forces is predicted by simulations (Fig. 2a, down; Supplementary Fig. 2e,f). To validate our force simulations, and to demonstrate the existence of repulsive forces in certain regions, we carried out force measurements on a test object (1.5 μm sphere of polystyrene containing superparamagnetic nanoparticles) scanned above the array of micro-magnets, in the presence of an in-plane field (see Methods). Repulsive forces were indeed measured, and good agreement was achieved with simulations (Supplementary Fig. 2g). If the UMLs were free to move, they would follow the force lines and thus move towards the micro-magnets (Figs 1i and 2a; Supplementary Fig. 2f). UMLs cannot aggregate or move inside a cell, since they are 200 nm in diameter, and thus trapped within the smaller mesh of the cytoskeleton which generally is of the order of 70 nm (see Methods)^36^. The magnetic force acting on the cytoskeleton produced by the UML–micro-magnet interactions is thus transmitted by the cytoskeleton in which UMLs are trapped, to the overall cell. Such forces dominate on the most apical part of the cell (see Methods), and thus dilate UML-loaded cells apex below and in front the pillars, while they compress those below and adjacent to the pillars along the dorsoventral y axis, forces being negligible along the antero-posterior *x*-axis (Figs 1i and 2a and Supplementary Fig. 2f). The application of the external magnetic field in pulsed mode, with a period comparable to the *sna*-dependent period of apex pulsations (Fig. 1b–g), thus results in soft constriction and dilation pulsatile forces on mesoderm tissue cells in the vicinity of the UML-loaded cells (Figs 1i and 2a). The apical constricting force applied to one UML in one cell was estimated to be of the order of 2 pN, leading to a force applied to one cell apex of 0.5±0.2 nN (Fig. 2a; Supplementary Fig. 2d–g with Methods).

To test our ability to quantitatively mimic *snail*-dependent pulsations in magnetically loaded embryos using a soft micro-magnet array coupled with a pulsating magnetic field, we first studied the ventral midline of *sna* RNAi *twi* RNAi-injected Spider–GFP embryos loaded with UML. These lack both active *sna-*dependent pulsations^31^, and the reactive Fog mechanosensitive pathway required for apical stabilization of Myo-II, downstream of *twi*^18^. In response to the pulsed localized field gradient forces, we observed the generation of individual cell apex size pulsations in the vicinity of the micro-magnets (Fig. 2b; Supplementary Movies 1–3). These pulsations quantitatively mimicked and rescued the *sna*-dependent pulsations (Fig. 2b–d), with a spectral signature showing a characteristic mean period of 138±28 s and a mean amplitude of 17±4% (Fig. 2e–g). This signature is comparable to the values of 126±22 s and 22±2%, which are characteristic of *sna*-dependent pulsations (Figs 1d,g and 2g).

These observations demonstrate the quantitative mimicking of individual cell *snail*-dependent mechanical pulsations within the multicellular mesoderm of the developing embryo, in a fully biochemically independent process, by the application of pulsating magnetic field gradient forces on UML-injected epithelia.

### Mechanical induction of medio-apical stabilization of Myo-II

We then applied pulsating localized magnetic field gradient forces to Spider–GFP; Myo-II–mCherry embryos injected with both blue fluorescent liposomes (Supplementary Fig. 3b) and *sna* RNAi only, starting 2 min before the end of cellularization, so as to mimic *sna*-dependent pulsations in the WT.

**Figure 3:**
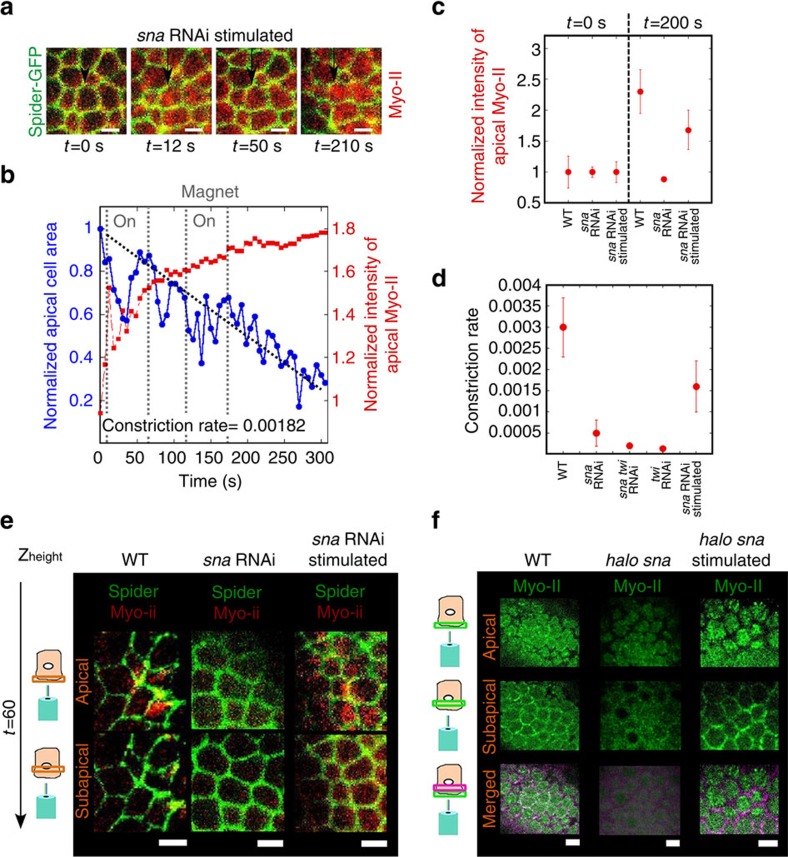
Rescue of medio-apical Myo-II in *sna*-defective embryos by mechanical pulses. (**a**) Dynamical imaging of apex size (Spider, Gilgamesh in green) and accumulation of Myo-II (1 μm below, merged in red) in *sna*RNAi-injected embryos in magnetically stimulated embryos mimicking *sna*-dependent pulsations in mesoderm cells. Representative of *n*=3 of the *N*=5 embryos (*P*=0.015, exact Fisher test). (**b**) Quantification on one representative cell of **a**. (**c**) Quantification of the apical accumulation of Myo-II at 200 s compared with 0 s in the WT, *sna* RNAi and *sna* RNAi magnetically stimulated. *N*=6 embryos for each condition (*P*<10^−3^ for both *sna* RNAi versus WT and *sna* RNAi magnetically stimulated). (**d**) Constriction rate in WT (*N*=6), versus *sna* RNAi-injected (*N*=6, *P*=0.004), *sna* RNAi *twi* RNAi-injected (*N*=6, *P*=0.02), *twi* RNAi (*P*=0.002) and *sna* RNAi magnetically stimulated (*N*=4, *P*=0.03) embryos. Scale bars, 5 μm. *P* values are calculated from the Mann–Whitney test, and error bars are s.e. (**e**) Spider (Gilgamesh, in green) and Myo-II (in red) cortical submembrane and junctional apical locations in the mesoderm cells of WT, *sna* RNAi and *sna* RNAi magnetically stimulated embryos, at *t*=100s. (**f**) Myo-II (in green) cortical submembranar and junctional apical locations in the mesoderm cells of WT, *halo sna* and *halo sna* magnetically stimulated embryos, at *t*=100 s. Representative of *n*=8, *n*=8 (*P*<10^−3^, exact Fisher test) and *n*=6 embryos (*P*=7 × 10^−3^) respectively, of the *N*=8 embryos observed in each case.

Focusing on Myo-II–mCherry, we found a medio-apical accumulation of Myo-II, with stable accumulation initiated after a time of 50 s (Fig. 3a,b; Supplementary Fig. 3a–c), rescued at 74% of the WT value at 200 s (Fig. 3c; Supplementary Fig. 4). Such Myo-II accumulation does not occur in non-pulsating *sna* RNAi-injected embryos, nor in embryos injected with UML and *sna* RNAi but not treated to magnetic stimulation (Figs 1e,f; Supplementary Fig. 5; Methods), nor in mechanically stimulated *sna* RNAi *twi* RNAi-injected embryos (Fig. 2b; Supplementary Fig. 6a,b). Interestingly, one pulse was sufficient to trigger the response in *sna* RNAi-injected embryos (Supplementary Fig. 6c).

Looking at both the apical submembrane and junctional planes, we observed that magnetic stimulation led to the rescue of stable medio-apical localization of Myo-II, beginning 50 s after the initiation of stimulation, both in junctions (co-localization with Gilgamesh at cell–cell contact in orange) and medio-apically (in red; Fig. 3e), followed by stable collective constriction (Supplementary Movies 4–6). Both sub-apical junctional and medio-apical submembranar localization of Myo-II mechanical rescue was also observed in *halo sna* Myo-II-GFP mutants (Fig. 3f), in which the *halo* phenotype allowed us to select homozygous mutants of *sna a priori* during cellularization and before gastrulation (Supplementary Fig. 1c)^28^.

Focusing on Spider–GFP, we found that magnetic stimulation quantitatively mimicked the first phase of *sna*-dependent pulsations (Fig. 3b). In contrast to *sna* RNAi *twi* RNAi-injected embryos, in which stable collective constriction and invagination were not triggered (Fig. 2d; Supplementary Movies 7 and 8; Supplementary Fig. 6a,b), the pulsatile phase of 150±12 s, comparable to the 170±10 s of the WT, was followed by stable apical constriction of cells, with the constricting rate rescued at nearly 50% of the WT (Fig. 3b,d). Associated medio-apical accumulation of Myo-II preceded stable apical constriction of mesoderm cells by 100±12 s (Fig. 3b), a value also consistent with the 110±10 s separating the two events in the WT (Fig. 1c)^28^.

**Figure 4:**
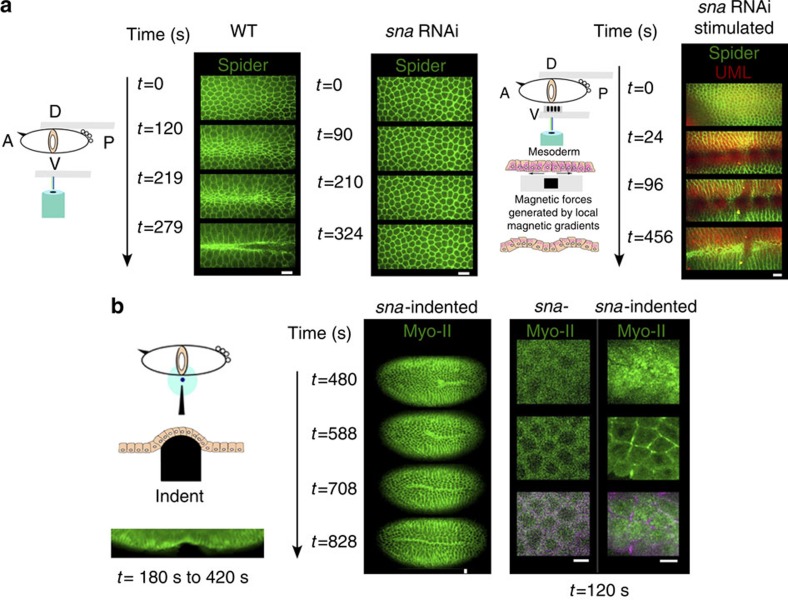
Rescue of mesoderm invagination in *sna*-defective embryos by mechanical pulses. (**a**) Coordinated apical constrictions in Spider–GFP followed by mesoderm invagination in the WT, all of which are impaired in *sna* RNAi-injected embryos (*n*=6 on *N*=6 injected), are rescued (constrictions initiating at 96 s and mesoderm invagination at 456 s) after the initiation of magnetic stimulation of *sna*-dependent pulsations in *sna* RNAi-injected embryos (UML in red). Representative of *n*=8 of the *N*=13 embryos observed (full invagination (*n*=5), partial invagination (*n*=3), *P*<10^−3^, exact Fisher test, considering the *N*=12 *sna*RNAi-injected embryos of Figs 1e and 4a that do not invaginate). The shadow line is a vitelline membrane folding independent of the process. (**b**) Mechanical rescue and propagation along the mesoderm of apical stabilization of Myo-II and mesoderm folding in *sna* mutant embryos by local indent: *sna-Myo-II-GFP* embryo indented 3 min after the end of ventral cellularization, and 4 min before the movie initiation and rotated 90°. Representative of *n*=19 of the *N*=22 embryos observed (10 total invaginations, 9 partial invaginations, *P*<10^−3^, exact Fisher test considering that none of the *sna* mutants invaginate). Zoom on the mesoderm of the indented *sna-Myo-II-GFP* embryo. Representative of *n*=4 of the *N*=4 embryos observed in each case (*P*<10^−3^, exact Fisher test, with 3 total invaginations, 1 partial invagination). Observations are realized with a × 20 objective. Scale bar, 5 μm.

Thus, the magnetically induced quantitative mimicking of the *sna*-dependent pulsations that rescue medio-apical accumulation of Myo-II in *sna* embryos, also rescued stable medio-apical constriction and mesoderm invagination from both the *sna* RNAi and *halo sna, sna-*defective phenotype (Fig. 4a; Supplementary Movies 4–6 and 9–11; Supplementary Figs 3a–c,e, 7).

We then checked, in time lapse *in vivo*, that a simple mechanical indent of 20 cells by a micromanipulated needle rescued both the apical accumulation of Myo-II and mesoderm cell constriction in *snail* mutants, initiating at the location of the indent, then propagating all along the mesoderm at a velocity of 0.29 μm s^−1^ (Fig. 4b; Supplementary Movie 12). This was confirmed in *halo sna* mutants (Supplementary Fig. 3d), with a lower efficiency than in the *sna* indented and in the *halo sna* magnetically rescued embryos (Fig. 4b Fig. 3f), presumably reflecting the cumulative perturbing effect of the *halo* genetic mutation and mechanical indent, two non-physiological parameters. Combined with the 150 s of the morphologic contractile response to magnetic deformation (Fig. 3b), one predicts a range of passive deformation by a minimum of 20 constricting cells initially deformed by the indent able to activate constriction of 0.29 × 150=45.5 μm. This represents 8.7 neighbouring cells of 5 μm size. In a linear approximation, this predicts that three cells but not two would be able to deform enough the neighbouring cells to trigger the stable constriction of the nearest-neighbour cells.

Such rapid autonomous propagation of active constrictions confirms that apical constrictions are mechanically activated in response to physiologically constricting neighbouring domains, rescued from the *snail* mutant phenotype.

These results show that the mechanical cues generated by cell pulsations allowed by *sna* expression are sufficient to trigger the coordinated apical accumulation of Myo-II leading to coordinated apical constriction and to mesoderm invagination.

### Medio-apical Rok/Myo-II Fog-dependent mechanical stabilisation

**Figure 5:**
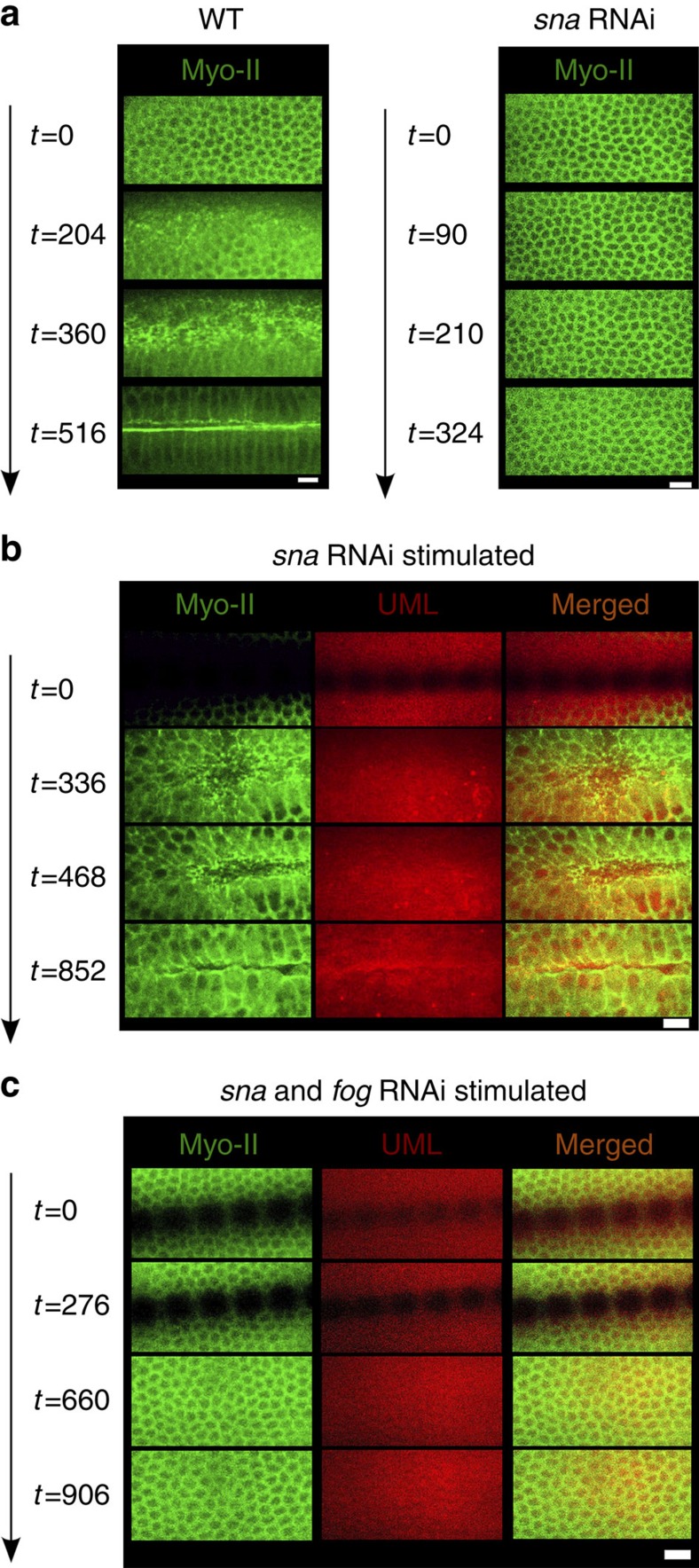
Rescue of medio-apical Myo-II by *sna*-like mechanical pulses is Fog-dependent. (**a**) Myo-II apical accumulation, followed by mesoderm invagination in the WT, all of which are impaired in *sna* RNAi-injected embryos (*N*=6 in each case, *P*=2 × 10^−3^, exact Fisher test). (**b**) Apical accumulation of Myo-II and mesoderm invagination after the initiation of magnetic stimulation of *sna*-dependent pulsations in *sna*RNAi-injected embryos (Myo-II in green, UML in red). Representative of *n*=6 of the *N*=6 *sna* RNAi embryos (*P*=2 × 10^−3^, exact Fisher test, Myo-II apical accumulation (*n*=6), full invagination (*n*=4), partial invagination (*n*=0)). (**c**) Sqh-GFP UML-loaded embryos injected with Sna RNAi and Fog RNAi and positioned close to the micro-magnet array, the ensemble being submitted to a magnetic field that is pulsed at a rate that mimics the rate of *sna*-dependent pulsations. Representative of *n*=7 of *N*=8 embryos (*P*=5 × 10^−3^, exact Fisher test). The micro-magnet array was removed after 336 s, so as to image the mesoderm directly above the individual micro-magnets. Scale bars, 10 μm.

The rescue of apical accumulation of Myo-II leading to constriction and mesoderm invagination by an indent of *sna* mutants with a needle was suggested to be induced by the mechanical blocking of Fog endocytosis due to membrane strain-induced effects, thereby leading to the over-activation of the downstream of the Fog signalling pathway. Indeed, rescue was found to depend on the expression of the Fog protein^18^. Consistent with the fact that Fog is expressed downstream of Twist^37^, we did not rescue apical accumulation of Myo-II, nor collective constriction nor mesoderm invagination in *twi*RNAi *sna*RNAi Myo-GFP embryos submitted to micro-magnet induced mechanical stimulation mimicking *sna-*dependent pulsations (Fig. 2b,d). Only a partial apical constriction deformation of several mesoderm cells was observed due to local pulsatile magnetic stimulations, thus mesoderm invagination did not occur (Supplementary Fig. 6a,b). We then submitted *sna*-defective embryos injected with Fog RNAi, to the magnetic stimulation that generated *sna*-dependent mimetic apex pulsations in *sna* RNAi-injected Myo-GFP UML-loaded embryos. These embryos showed no detectable rescue of apical accumulation of Myo-II (increase of apical Myo-II to 1.04±0.14 compared with before stimulation), nor mesoderm cell constriction nor mesoderm invagination observed in both the WT and the stimulated *sna* RNAi-injected embryos (Fig. 5a–c; Supplementary Movie 13). Similar results were consistently observed with Rok, for which medio-apical relocalization is required for Myo-II medio-apical relocalization^38^ (Supplementary Fig. 8a–c; Supplementary Movies 14–17).

These results show that the Fog protein, which is downstream of Twist, is required in the *sna-*dependent mechanically induced medio-apical polarization of Rok that leads to mechanically triggered medio-apical accumulation of Myo-II, apical constriction and mesoderm invagination.

### Mesoderm apex constriction triggers endoderm apical Myo-II

**Figure 6:**
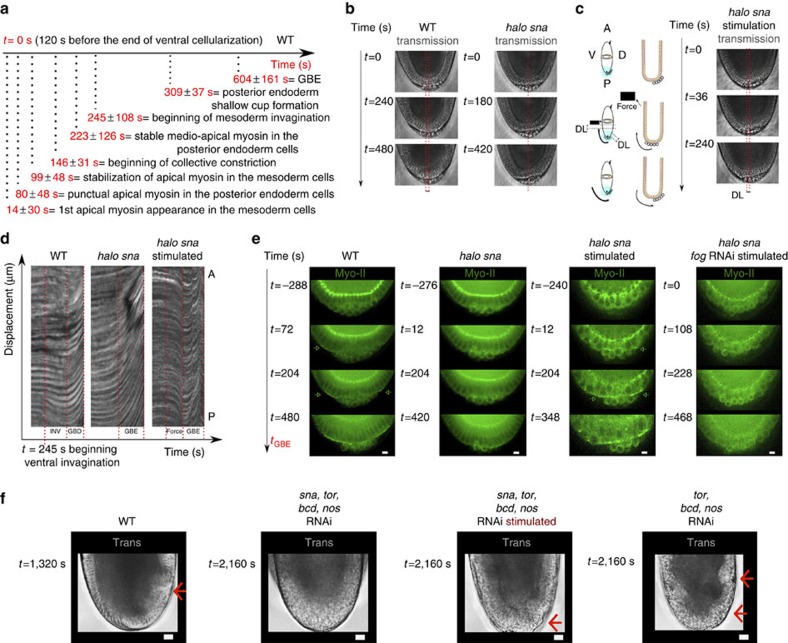
Fog-dependent induction of endoderm apical Myo-II by mesoderm invagination. (**a**) Timing of the sequence of morphogenetic events at gastrulation. (**b**) Stretching of posterior endoderm cells by mesoderm invagination in the WT, followed by compression by GBE. Such stretching is shown to be absent in *halo sna* mutants. Representative of the *N*=6 embryos observed in each case. (**c**) Mechanical rescue of posterior endoderm stretching by highly localized magnetic interactions with UML-loaded mesoderm in *halo sna* mutants. (**d**) Chemographs of the posterior endoderm of WT, *halo sna* and magnetically stretched *halo sna*. Representative of the *N*=6 embryos observed in each case. (**e**) Distribution of Myo-II-GFP in sqh-GFP, *halo sna* sqh-GFP, *halo sna* sqh-GFP magnetically stretched posterior endoderm, and Fog RNAi-injected *halo sna* sqh-GFP magnetically stretched posterior endoderm. Representative of the *N*=6 WT embryos, the *N*=6 *halo sna* embryos *P*=2 × 10^−3^, exact Fisher test), the *N*=14 *halo sna* stretched embryos (*P*<10^−3^) and the *N*=7 *halo sna* Fog RNAi-stimulated embryos (*P*<10^−3^). Scale bars, 5 μm. (**f**) Invagination (red arrow) in the posterior endoderm of non Rnai-injected (representative of the *N*=6 embryos observed), *sna, bcd, nos, tor* Rnai-injected (representative of the *N*=4 embryos observed, *P*=2 × 10^−3^), *sna, bcd, nos, tor* Rnai magnetically stretched posterior endoderm (representative of *N*=5/8 embryos, *P*=0.02), and *bcd, nos, tor* Rnai embryos (*N*=4, *P*=0.03). Scale bars, 10 μm.

Apical accumulation of Myo-II leading to epithelial invagination and folding was first identified in the posterior endoderm formation of gastrulating *Drosophila* embryos^1^. Strikingly, while Sna and Fog were found to be required for apical accumulation of Myo-II and proper invagination of the mesoderm, Fog, but not Snail, is expressed in the posterior endoderm^39^,^40^. On the other hand, stable apical accumulation of Myo-II in the posterior endoderm initiates at 223±126 s, after contractile stabilization in the mesoderm at 99±48 s (Fig. 6a,e). The accumulation is subsequently reinforced during mesoderm folding until mid-invagination, before the initiation of the germ-band extension morphogenetic movement of convergent extension (Fig. 6a,e). This correlates with the initiation of the posterior endoderm cell stretching observed in response to constriction and folding of ventral mesodermal cells^41^ (Fig. 6a,b,d). We thus tested the hypothesis that the mechanical strain dilations developed by mesoderm cell constriction and folding on the posterior endoderm epithelium activates the apical stabilization of Myo-II, in a Fog-dependent process.

We first characterized apical accumulation of Myo-II in posterior endoderm cells in non-invaginating *halo sna* mutants. In contrast to the WT, we found no ventral stretching of posterior endodermal cells as observed directly from the position of posterior pole germ cells, and confirmed in the kymographs of posterior endodermal cells (Fig. 6b,d). In contrast to the WT, no apical accumulation of Myo-II in endoderm cells was observed up to *t*=204 s (Fig. 6e), that is, during the equivalent time of the WT first stochastic and second collective constriction phases. We rescued the mechanical effect of stretching of posterior endoderm cells by mesoderm invagination in the *halo sna* mutant, by attracting UML-loaded posterior mesoderm cells with an array of hard (that is, permanently magnetized) micro-magnets of size 20 μm deposited onto a patterned Si substrate^42^, attached to a micro-manipulator, 6 min before the end of cellularization (Fig. 6c,d). We observed a rescue of the apical accumulation of Myo-II in both the ventral and dorsal endoderm beginning at *t*=12 s, namely, 4 min after the initiation of stretching, and this was re-enforced between *t*=204 s until *t*=348 s (Fig. 6e). The same dynamics of rescue were observed when the stretching was generated from the dorsal side of the embryo, that is, independently of any rescue of mesoderm invagination by magnetic manipulation in the mesoderm, thereby excluding any mechanical-independent developmental rate effect associated to mesoderm formation *per se* (Supplementary Fig. 9). Given the nearly 3 min delay between the initiation of the magnetic force in the mesoderm and the observation of deformation in the posterior endoderm (Fig. 6d), the timescale of Myo-II apical stabilization rescue is nearly 1 min. This is consistent with the observation of a response initiating nearly 1 min after the first appearance of apical myosin in the mesoderm of WT (Fig. 6a,e), as well as with the 1 min timescale of the Myo-II response to magnetically induced and the <2 min timescale indent induced, mechanical strains observed in the mesoderm (Figs 3b and 4b, right, respectively). Such rescue was impaired in embryos previously injected with Fog RNAi (Fig. 6e).

**Figure 7:**
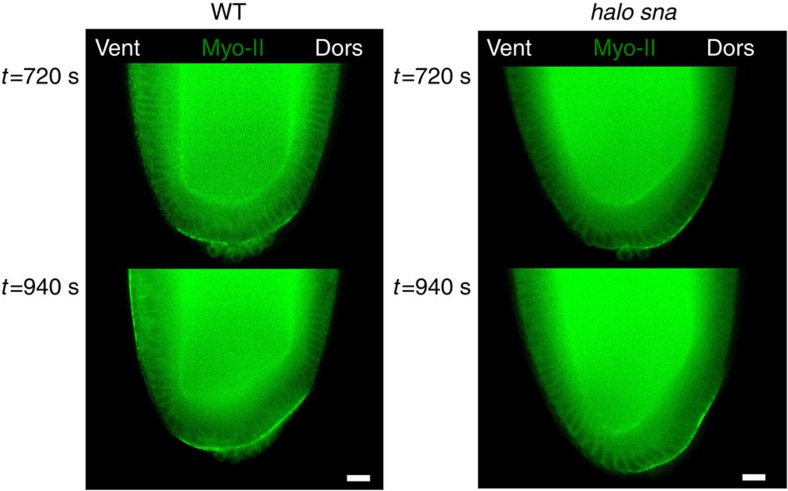
Distribution of Myo-II-GFP in *halo sna* at germ-band extension initiation. Representative of the *N*=6 WT embryos and the *N*=6 *halo sna* embryos (*P*=2 × 10^−3^, exact Fisher test). *halo sna* invaginate their posterior endoderm (later stages, not shown), as do *sna* mutants. Scale bars, 5 μm.

Germ-band extension (GBE), which compresses posterior pole cells 8 min after they have been stretched by mesoderm invagination (Fig. 6a), could subsequently also participate in the posterior endoderm apical accumulation of Myo-II and invagination in a *sna*-independent process, after stretching due to mesoderm invagination. We thus tested the role of the *sna*-dependent mesoderm invagination induced mechanical activation of apical relocation of Myo-II, in endoderm invagination under *bcd, nos, tor* germ-band defective conditions^43^. In *sna* and *bcd, nos, tor* RNAi-injected embryos, lacking both mesoderm invagination and germ-band extension, no invagination was observed in the posterior endoderm (Fig. 6f). The magnetically induced mimicking of posterior endoderm cell stretching by *sna*-dependent mesoderm invagination rescued shallow cup formation, that is, the initiation of endoderm invagination^41^, in these embryos (Fig. 6f). Consistently, *bcd, nos, tor* RNAi-injected embryos showed shallow cup formation initiation, associated with *sna*-dependent mesoderm invagination (Fig. 6f; Supplementary Movies 18 and 19; Supplementary Fig. 10e). Note that here the initiation of endoderm invagination is less pronounced than in the WT, possibly due to the lack of an additional germ-band extension buckling driving force in the *bcd, nos, tor* RNAi-injected embryos. Similarly, *bcd*, *nos, tsl* germ-band defective mutant embryos are also known to initiate posterior endoderm invagination through a shallow cup formation^44^, as observed here with posterior folding and the cup shaped yolk–epithelium interface, respectively—two red arrows in Fig. 6f. Finally, *sna*-defective embryos showed apical accumulation of Myo-II and the initiation of invagination associated to *bcd, nos, tor*-dependent germ-band extension at later stages after 600 s (Fig. 7).

These experiments demonstrate the Fog-dependent mechanical induction of posterior endoderm apical stabilization of Myo-II and invagination initiation triggered by the mechanical strains developed by mesoderm constriction and invagination, which is subsequently reinforced by morphogenetic movement of GBE.

## Discussion

The search for the biochemical principles regulating epithelial cell folding leading to tissue invagination is a long-standing challenge in developmental biology^30^. Specific interest has been focused on the endoderm and mesoderm primary invaginations of gastrulation, which correspond to the very first morphogenetic movements of embryogenesis in many distinct species^45^. One of the best genetically characterized morphogenetic movements is mesoderm invagination of early *Drosophila* embryos, which is regulated by the interplay between the two pathways established by the expression of the first zygoticaly expressed ventral genes *sna* and *twi*. However, while the expression of *sna* is known to be required for the *twi*-dependent stable medio-apical accumulation of Rok and Myo-II, mesoderm cell constrictions and invagination, the underlying mechanism of the interplay between the two pathways remains poorly understood^26^,^31^,^38^,^46^,^47^. Because *sna* expression leads to cell apex size pulsations through a process that remains to be precisely characterized, thereby introducing mechanical strains in mesoderm cells, it was experimentally (by using a simple but non-physiological indent mechanical perturbation) and theoretically (by using simulations) suggested that *sna* could activate the Rho pathway leading to apical accumulation of Myo-II, through a downstream of *twi* Fog-dependent mechanotransductive process^18^,^48^. This hypothesis was in line with the finding of a *twist*-dependent increase of probability of having stable apex constriction in the cells neighbouring already constricting cells^49^.

The results presented above demonstrate that quantitative mimicking of *sna*-dependent pulsations in the absence of *sna* expression, leads to (i) the rescue of medio-apical stabilization of Rok, (ii) apical and medio-apical stabilization of Myo-II, (iii) coordinated stable apical constriction and mesoderm invagination. All these processes, absent in *sna* mutants, are rescued on the timescale of a few minutes. In addition, we show that the rescue of these WT phenotypes from the *sna* phenotypes is Fog-dependent. This demonstrates the existence of a Fog-specific mechanotransductive activation of medio-apical polarization of Rok, leading to apical stabilization of Myo-II by the mechanical strains resulting from *sna*-dependent pulsations in the mesodermal cells of *Drosophila* embryos, on the timescale of a few minutes.

Interestingly, the fact that the rate of constriction rescue is nearly two times lower in the absence of *sna* than the constriction rate of the WT, and has a lower intensity of Myo-II accumulation at the timescale of invagination initiation (>300 s), possibly reflects the lack of one of the receptors of Fog (that is, Mist) in *sna* mutants (Fig. 3b,c)^50^,^51^. Nevertheless, these less intense responses to mechanically induced pulsations led to mesoderm invagination rescue in *sna* mutants, with similar dynamics to that of the WT, showing that *sna*-dependent pulsations are sufficient to trigger the medio-apical stabilization of Myo-II required for mesoderm invagination.

Invagination specifically requires the coordinated constriction of apexes. Coordination of individual cell behaviour can be generated in response to a common external biochemical morphogenetic field, such as the expression of the Fog secreted signal protein^46^. However, ectopic expression of Fog in *sna* mutants, as well as Fog overexpression in the mesoderm of *sna*-defective embryos, do not lead to apical accumulation of Myo-II, nor apical constriction nor mesoderm invagination^18^,^39^. Coordination of individual cell behaviour can be achieved if the behaviour of a given cell activates the same behaviour in its neighbouring cells though cell–cell interactions, thereby leading to correlated collective effects. In the specific case dealt with here, the individual cell behaviour is apical constriction, and is thus mechanical in nature. It was suggested that the mechanical activation of cell constriction by a neighbouring constricting cell can drive collective and coordinated constriction^48^,^52^. Here we propose that *sna*-dependent pulsations represent a phase of unstable autonomous cell apex constrictions generating cell–cell mechanical interactions, which trigger a transition to a phase of coordinated apical constriction. Such a transition emerges from a Fog-dependent mechanotransductive activation of the stable constriction of cells mechanically deformed by their neighbouring pulsating constricting cells.

Invagination of both the mesoderm and posterior endoderm is Fog-dependent and induced by a process of apical accumulation of Myo-II^1^,^41^,^46^. Strikingly, we show here that the initiation of both apical Myo-II accumulation in the mesoderm and invagination in the posterior endoderm require the expression of *sna* (Fig. 6e), while the posterior endoderm does not express *sna*. However, we found that *sna* is not biochemically required in itself for the medio-apical accumulation of Myo-II leading to mesoderm invagination, rather the mechanical strains developed by *sna*-induced pulsations are needed. Similarly, we show that the triggering of apical accumulation of Myo-II and posterior endoderm invagination is induced by the mechanical strain dilations developed by the constriction of mesoderm cells, on the timescale of a few minutes. We also establish the conservation of the Fog-dependent mechanotransductive induction of Myo-II apical accumulation by mesoderm invagination, in the posterior endoderm. Thus, the fact that *sna* is required to mechanically trigger apical accumulation of Myo-II and mesoderm invagination implies that *sna* expression is also required to initiate posterior endoderm invagination, even though its expression is not required in posterior endoderm cells. As a consequence, mesodermal *sna* interacts with posterior endodermal Fog, over the distance of five cells separating the two tissues, through mechanical cues, known to propagate rapidly and over long distances across materials. In addition, we find that mechanical induction of Myo-II apical accumulation and invagination in the posterior endoderm is subsequently reinforced by the morphogenetic movement of GBE.

**Figure 8:**
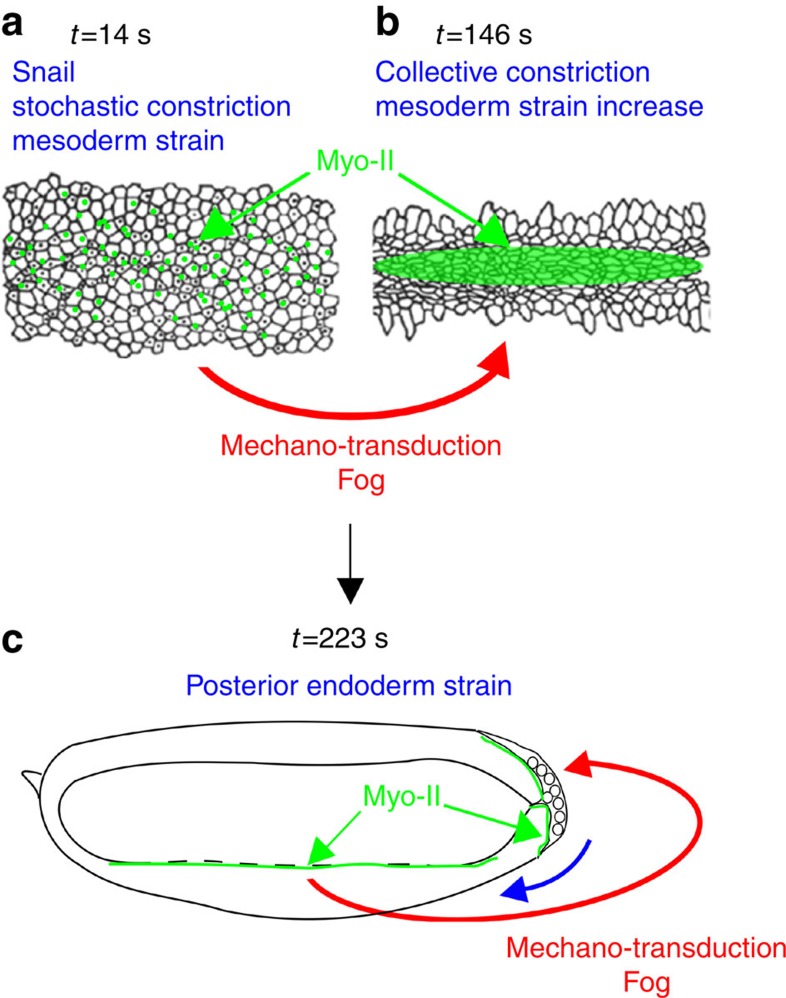
Meso-endoderm mechanotransductive cascade of Myo-II apical stabilization. (**a**) *Sna*-dependent stochastic fluctuation of mesoderm apex size (**b**) activates the medio-apical accumulation of Myo-II in a Fog-dependent mechanotransductive process leading to coordinated collective constriction of the cell apex (initiating nearly 130 s after the initiation of pulsations), then mesoderm invagination. (**c**) In turn, mesoderm constriction generates a strain deformation of posterior endoderm cells (blue array) that activates the apical stabilization of Myo-II required for endoderm invagination nearly 80 s after mesoderm collective constriction initiation, that is subsequently reinforced during mesoderm invagination, in a Fog-dependent mechanotransductive pathway. Adapted from the figures of morphogenetic movements of Sweeton *et al*.,^26^ *Development*, 1991 and Nüslein-Volhard & Wieschaus, *Drosophila* a practical approach, 1896.

Embryonic development has long been considered as the product of a cascade of developmental gene expression, with characteristic timescales ranging from 10 min to hours for each individual step of the cascade^29^,^30^. Here we demonstrate that the Fog-dependent mechanotransductive induction of medio-apical polarization of Myo-II regulates a self-inductive non-transcriptional rapid cascade of active morphogenetic movements at the shorter timescale of minutes. *Sna*-dependent mesoderm apex pulsations trigger the Fog-dependent medio-apical stabilization of Myo-II that activates the coordinated constriction of apexes generating mesoderm folding and invagination within a few minutes. Such coordinated constriction and mesoderm invagination in turn activates the Fog-dependent apical accumulation of Myo-II initiating the next morphogenetic movement of posterior endoderm invagination, which also occurs within a few minutes (Fig. 8). Snail and Fog expression being the result of the biochemical cascade of differentiation, we thus show the coexistence at different timescales, and interactions between a slower differentiation gene expression biochemical cascade and a rapid self-inductive mechanotransductive cascade. Here we thus reveal that, during and patterned by the expression of developmental differentiation genes, mechano-proteic mechanotransductive interplay drives a rapid self-inducing cascade of morphogenetic movements in early *Drosophila* gastrulation.

## Methods

### Fly strains

Oregon R was taken as the WT. Male and female of any age were used. Myo-II-GFP (*sqh-GFP*) was provided by Roger Karess, Myo-II–mCherry Gilgamesh–GFP (*sqh–mCherry spider–GFP*), *halo sna* Myo-II-GFP (*halo snailIIG05/CyO, sqhp–sqh::GFP*) were provided by Adam Martin^28^, *sna* Myo-II-GFP (*snailIIG05/CyO, sqhp–sqh::GFP*) were produced by crossing *halo snailIIG05/CyO, sqhp–sqh::GFP* with *snailIIG05/CyO*, rok-GFP (*w,Ubi-Rok-GFP; et w;; Ubi-Rok-GFP/TM6*)^21^ and Gilgamesh–GFP (s*pider–GFP)*^53^ were provided by Yohanns Bellaïche. Stocks were maintained at room temperature (22 °C), and experiments and observations have been done at 24 °C.

### RNAi

Three RNAi sequences a gene were designed and produced by Eurogentec

Sna RNAi (1,481–1,503 S 5′: CAGCUAUAGUAUUCUAAGU dTdT; AS 3′: ACUUAGAAUACUAUAGCUG dTdT, 340–362 S 5′: GGAACCGAAACGUGACUAU dTdT; AS 3′: AUAGUCACGUUUCGGUUCC dTdT, 972–994 S 5′: CCGAGUGUAAUCAGGAGAA dTdT; AS 3′: UUCUCCUGAUUACACUCGG dTdT).

Twi RNAi (928–950 S 5′: GCACCAGCAACAGAUCUAU dTdT; AS 3′: AUAGAUCUGUUGCUGGUGC dTdT, 2,061–2,083 S 5′: GUCACGCUUUCCAUAUAUA dTdT; AS 3′: UAUAUAUGGAAAGCGUGAC dTdT; 1–1,993 S 5′: CGGAUCAGGACACUAUAGU dTdT; AS 3′: ACUAUAGUGUCCUGAUCCG dTdT).

Fog RNAi (98–12,520 S 5′: CUGGCCAGAUUAGGUAGAU dTdT; AS 3′: AUCUACCUAAUCUGGCCAG dTdT, 7,054–7,076 S 5′: CUGAACACCUCACAUAAUA dTdT; AS 3′: UAUUAUGUGAGGUGUUCAG dTdT, 3,537–23,559 S 5′: CGUGUGAAGUUGAUUUCAA dTdT; AS 3′: UUGAAAUCAACUUCACACG dTdT).

The sequences were designed to not interfere between themselves, and with *myo-II*, *rhogef2*, *EB1*, *cta*, *T48*, *traf4*, *jupiter*, *arm*, *src42A, src64B, shg, khc, klc*.

Bicoïd-RNAi (3,592–3,614 S 5′: CUACUCUACUCGGGUAAAU dTdT, 699–721 S 5′: CACACCGACAAUCAGUAAU dTdT, 502–524 S 5′: GCGAAAUCUUCUAUUAUCU dTdT).

Nanos-RNAi (1,673–1,695 S 5′: GAGCUUCCAAUUCCAGUAA dTdT, 163–185 S 5′: GCGAAUACUUCAGUUGAAU dTdT, 726–748 S 5′: CGCAGCAGGUAAGAAGAAA dTdT).

Torso-RNAi (771–793 S 5′: GUAGUCAUCAAGACCUAAU dTdT, 3,327–3,349 S 5′: GUCAGAGCGAUGUGUAAGU dTdT, 2,707–2,729 S 5′: CCUCACUCGAAUAACCAAU dTdT).

The effect of RNAi on protein levels was all validated in western blots (see just below), except for Torso, for which no antibody was available in the community (Supplementary Fig. 10a–d).

### Western blots

Protein extracts from *Drosophila* embryos were prepared by collecting dechorionated and injected or Oregon stage 5 embryos and placing them into 40 μl lysis buffer (50 mM Tris pH 8.0, 150 mM NaCl, 0.5% Triton X-100, 1 mm MgCl_2_ with added Complete Protease inhibitor Cocktail without EDTA (Roche) on ice). Embryos were homogenized on ice using a hand homogenizer from VWR with pestle for eppondorf tubes. The homogenate was stored afterwards at −20 °C until further use. Homogenate was then centrifuged for 2 min at 5,000 r.p.m. and 4 °C. A measure of 30 μl of the supernatant was stored at −20 °C. Protein concentration was measured at 280 nm using the NanoDrop ND-1000 Spectrophotometer system. For western blot sample loading, the equivalent of 10 μg was loaded for RNAi-treated embryos and for WT 100. SDS–polyacrylamide gel electrophoresis and blotting were carried out using the Bio-Rad western blot system. Nitrocellulose membranes were blocked using TBST 5% bovine serum albumin (BSA) for a minimum of 2 h and then incubated with the primary antibodies overnight in TBST 5% BSA. Primary antibodies were used in the following dilutions in TBST 5% BSA: antibodies used were guinea pig a-Sna donated by Eric Wieschaus (Princeton University, USA, dilution 1:2,000)^54^, rabbit a-Twi donated by Siegfried Roth (Köln University, Germany, dilution 1:1,000)^55^, rabbit a-Fog donated by Eric Wieschaus (Princeton University, USA, dilution 1:400)^46^, rabbit a-Nanos donated by Dahua Chen (Beijing University, China, dilution 1:4,000)^56^ and rabbit a-Bicoid donated by Steve Small (New-York University, USA, dilution 1:500)^57^. Secondary antibodies were horseradish peroxidase-coupled anti-rabbit 1:1,000 (Cell Signaling 7074S) and anti-guinea pig 1:4,000. After incubation of blots with Pierce ECL Plus Western Blotting Substrate (Thermo Scientific) for 5 min, blots were imaged using Amersham Imager 600. For the lading control, blots were stripped by incubating the blot membrane in SDS 2%, Tris HCl pH 6.8 63 mM, 8‰ ß-mercaptoethanol under the fumehood and slight agitation for 40 min. After washing under running water for 2 h, blots were blocked overnight in TBST 5% BSA. Primary antibody mouse against ß-actin (AC-15, sc-69879, Santa Cruz Biotechnologies) was added in TBST 5% BSA (1:1,000) and incubated overnight. Horseradish peroxidase anti-mouse secondary antibody (Cell Signaling 7076S) was added in a 1:1,000 dilution on TBST. Precise protein molecular weights were calculated from their amino-acid sequence, from the Uniprot database for the annotated proteins ( http://www.uniprot.org/): Snail P08044: 42955.33 D; Twist P10627: 54421.83 D; Fog P40795: 76109.92 D; Bicoid P09081: 54511.22 D; Nanos P25724: 43427.78 D; Actin 5c P10987: 41587.51 D. Gel images were analysed using the gel analysis tool in ImageJ, with all bands analysed normalized to respective actin controls.

All experiments were performed on a set of nearly 20 embryos injected at stage 4, and analysed at cellularization stage, randomly ranging from early stage 5 to late stage 5. The *sna* RNAi injection induced the specific decrease of 45% of Sna expression (with no decrease of Bicoid or Nanos expression as a control) as a mean value characteristic of the overall stage 5 (Supplementary Fig. 10a). This was sufficient to lead to the specific inhibition of mesoderm invagination at early stage 6 (Figs 1e,f and 3a). Given the delay between RNA degradation and protein degradation, this is consistent with the 60% decrease of* sna* RNA sufficient to inhibit apex pulsations and mesoderm invagination observed in reference^28^. *bicoid*-RNAi *nanos*-RNAi *torso*-RNAi injection induced the specific decrease of 34% of Bicoid and 34% of Nanos (with no significant decrease of Twist expression as a control; Supplementary Fig. 10b), as mean values characteristic of the overall stage 5, which together was sufficient to lead to the specific inhibition of germ-band extension at mid-stage 6 (Supplementary Fig. 10c). Note that the Torso antibody was not currently available (in the community) to test for Torso protein.

Twi RNAi induced the specific decrease of 46% of Twist expression as a mean value of the overall stage 5 (with no decrease of Bicoid expression as a control; Supplementary Fig. 10c), and Fog RNAi induced the specific decrease of 73% of Fog expression (with no decrease of Bicoid expression as a control; Supplementary Fig. 10d).

Thus, RNAi treatments let to a specific decrease of the order of 70% of the signalling protein Fog, of 40% of the targeted proteins Snail and Twist, and of the order of 30% on the Bicoid and Nanos proteins having a combining effect in the production of germ-band extension, during stage 5. Such quantitative gradation is consistently inversely related to the developmental time at which the RNAs producing the proteins are initially loaded into the embryo, with *bicoid* and *nanos* RNAs being maternally loaded from earliest times of embryonic development^58^,^59^, whereas *twist* and *snail* are zygotically expressed from stage 4, before the onset of cellularization^27^,^60^, and the strong zygotic expression of *fog* is characteristic of the later late-stage 5 (ref. 39). In addition, these mean values are representative of the overall cellularization stage 5 (that takes 45 min of development). Therefore, these are probably significantly underestimated compared with the values specifically characterizing embryos at the end of stage 5 that condition early stage 6 functional effects, which necessarily benefits from the largest amount of time for targeted protein degradation in the absence of renewal under RNAi conditions.

Un-cropped western blots can be found in Supplementary Fig. 11.

### Embryo preparation

Egg collections were performed on agar cups covered with grape-juice plates. Flies were allowed to lay eggs for 2–4 h at 25 °C before the plate was removed. During preparation, embryos were never compressed to avoid any process of mechanotransduction before controlled mechanical stimulation. In all experiments, *Drosophila* embryos were dechorionated with 50% bleach until the ‘antenna' disappear (nearly 40 s), then washed with water. Embryos were then glued on a coverslip covered with glue (double-sided tape soaked in heptane), ventral side up (for UML injections Fig. 2), ventral side down (for controls, Fourier transform, Figs 1 and 2) and laterally (for indent experiments and magnetic stimulation Figs 4 and 6). In case of injection, embryos were previously dried 7 min in the presence of salt. A few droplets of Halocarbon oil 27 were placed on the embryo to prevent drying and to allow observation. After injection, an area of 2.5 cm × 1 cm including the embryo was cut with a diamond point, turned upside down and glued on the piezo-electric system to observe the ventral side in reflection, and to allow controlled relative movements of the embryo with respect to the magnetic pattern with 100 nm resolution. For indent experiments, the coverslip with the glued embryo was put on the microscope stage, and the indenting needle was attached to the piezo-eclectic device.

### Ultra-magnetic nano-particle preparation

The synthesis of MNPs is described first. The aqueous suspension of MNPs was prepared using alkaline co-precipitation of FeCl_2_ (0.9 mol) and FeCl_3_ (1.5 mol) salts, according to Massart's procedure^61^. Superparamagnetic maghemite grains (γ-Fe_2_O_3_) were obtained by oxidizing 1.3 mol of magnetite with 1.3 mol of iron nitrate (boiling solution). After magnetic decantation, 2 l of distilled water and 360 ml of HNO_3_ 20% were added to the solution and the mixture was stirred for 10 min. Prepared maghemite nanoparticles were washed several times with acetone (3 × 1 l) and ether (2 × 500 ml) and suspended in water. Size sorting was performed by adding HNO_3_ (0.45 M) to the suspension followed by magnetic decantation. This operation was repeated with the deposit until suitable particle size was obtained. Sodium citrate (*n*_Fe_/*n*_Cit_=0.13) was added to the nanoparticles and the mixture was heated at 80 °C for 30 min to promote absorption of citrate anions on their surface. Citrated nanoparticles were precipitated in acetone and suspended in water. The volume fraction and average size of the maghemite grains were determined by fitting the magnetization curve of nanoparticles using the Langevin function. Particles of 9 nm diameter (s.d. *σ*=0.35, volume fraction of nanoparticles in the suspension *ϕ*=1.9%, specific susceptibility χ/*φ* of 15.5) were obtained. For UML preparation, the aqueous medium was removed using a Macrosep centrifugal device 30 kDa (PALL) and nanoparticles were suspended again in a buffer (0.108 M NaCl, 0.02 M sodium citrate and 0.01 M HEPES, pH=7.4).

The preparation of UMLs is described second. Solutions of 1,2-dipalmitoyl-*sn*-glycero-3-phosphocholine (DPPC), 1,2-distearoyl-*sn*-glycero-3-phosphocholine (DSPC), 1,2-distearoyl-*sn*-glycero-3-phosphoethanolamine-n-[(carboxy(polyethyleneglycol)2000](ammonium salt) (DSPE-PEG2000) and L-α-phosphatidylethanolamine-*N*-(lissamine rhodamine sulfonyl B) (ammonium salt) (Rhod-PE) in chloroform were purchased from Avanti Polar lipids, Inc. UMLs were prepared by the reverse-phase evaporation method established by Skoza *et al*.^62^ and modified according to a protocol previously described^34^. In brief, a mixture of DPPC/DSPC/Rhod-PE/DSPE-PEG2000 (84/10/1/5 mol%, 315 μl) was dissolved in 3 ml of diethyl ether (VWR) and 900 μl of chloroform (Carlo Erba Reagents). Thereafter, 1 ml of citrated MNPs dispersed in the buffer was introduced before sonication at room temperature for 20 min to produce a water-in-oil emulsion. Organic solvent was evaporated with a rotavapor R-210 (Buchi) at 25 °C until the gel phase disappeared. Liposomes were filtrated through a 450 nm filter and purified from nonencapsulated MNPs by magnetic sorting using a bulk magnet (Calamit Fe−Nd−B 150 × 100 × 25 mm). The operation was repeated three times every 2 h and liposomes were finally separated from the supernatant and recovered.

### Injection of UML and RNAi

An injector (Eppendorf Femtojet) was fixed on the microscope to inject magnetic particles or/and RNAi. A *x*,*y*,*z* piezo-electric system (Princeton Instruments) was mounted on the microscope to control the position of the embryo mounted on a coverslip relative to the injecting needles, the micro-magnets or the indent needle, with 100 nm resolution.

Using a Femtojet Eppendorf injector, ∼0.05 μl of 0.1 μm g^−1^ UML was injected into embryos along cell basal domain inside the ventral cells of the embryo from 40 to 20 min before the end of ventral cellularization. Injections were performed at constant pressure, with the visualization of UML labelled with Rhodamin observed in red in spinning disc. Before initiation of the *sna*-dependent pulsating phase, all blastoderm cells are under cellularization and are basally open to the yolk^41^. UMLs, composed of a high concentration of 9 nm Fe-oxide superparamagnetic nanoparticles (20% v-v) encapsulated in 200 nm fluorescently labelled phospholipid membranes^34^, were thus injected into the yolk below and all along mesoderm cells, 20–40 min before the end of cellularization (Fig. 1h; Supplementary Fig. 2a). At the end of cellularization, the embryo was turned 180° ventral to non-magnetized (that is, no external magnetic field applied) soft micro-magnets embedded in a 50 μm-thick transparent PDMS membrane (Fig. 1i; Supplementary Fig. 2a,b). Note that WT embryos injected with UML gastrulated normally (Supplementary Fig. 1b).

Approximately 0.05 μl of 0.1 μm g^−1^ UML and ∼0.05 μl of 0.1 μg μl^−1^ RNAi were injected from 80 to 60 min before the end of ventral cell cellularization except for injections including *bcd, nos* and *tor* Rnai having been injected as soon as possible, 2 h 30 min to 3 h before the end of cellularization. The UML and the RNAi were injected together inside the embryo along cell basal domain inside the ventral cells. In case of injection of two types of RNAi, embryos were injected from 100 to 80 min before the end of ventral cell cellularization.

### Fabrication and simulation of micro-magnets

Deep reactive ion etching was used to produce a linear array of Si pillars of cross section 10 × 10 μm^2^ and height 40 μm, separated by a distance of 20 μm, at the surface of a Si substrate. High-rate triode sputtering was then used to deposit a Ta (100 nm)/Fe (10 μm)/Ta (100 nm) trilayer onto the patterned Si substrate (the Ta buffer and capping layers serve to protect the Fe layer from reaction with the substrate and from oxidation, respectively). The thickness of Fe deposited on the sidewalls of the pillars is limited due to the directional nature of sputtering and to shadowing effects from neighbouring pillars. A 50 μm-thick layer of PDMS was spin-coated onto the structure and, after reticulation, the PDMS layer was pulled off the substrate in such a way that the embedded magnet-topped Si pillars break away from the substrate. The resultant structure consists of a PDMS membrane containing a linear array of soft micro-magnets of approximate cubic shape, just below the membrane surface. More details can be found in ref. 63.

A SHPM was used to measure the stray field pattern produced by the micro-magnets^64^. The micro-magnet array was positioned on top of a bulk NdFeB magnet, oriented in such a way as to produce a field that is comparable to that applied by the electromagnet during bio-experiments (in-plane, oriented perpendicular to the axis of the array of micro-magnets). The Hall probe was mounted at an angle of ∼23° with respect to the *y* axis, for experimental reasons, so that the measured signal had contributions from both the *z* and *y* field components (*B*_*z*_ cos23°+*B*_*y*_ sin 23°). The scan was made at a distance of roughly 10 μm above the micro-magnet array. Note that the field component measured is determined by the orientation of the probe and that it is not possible to measure the *y* component of the field pattern above the micro-magnets using our SHPM. Nevertheless, the good agreement observed between measured and simulated patterns validates our simulations of all field components.

Simulation of the stray magnetic field pattern produced by the micro-magnet array was performed. To estimate the force applied by the micro-magnet array on an UML, we need to know the magnetic field produced by it. This was measured using a SHPM, at a scan height of 10±5 μm, as shown and described above (Fig. 2a). We then simulated the field profile in an analytical approach using Matlab. We considered a linear array of seven cubes of Fe, of edge length 10 μm, separated by 20 μm, magnetized by an in-plane field, applied perpendicular to the axis of the array. The magnetization of the Fe micro-magnets was assumed to be given by the demagnetizing susceptibility so that *M=H*_appl_*/N*, where *N* is the demagnetizing field factor of the micro-magnet, which is taken to be 1/3. This gives *M*=0.18 × 3=0.54 T. The applied field used in the calculation was that produced by the bulk magnet used to magnetize the micro-magnets in the SHPM. The topologies of the field profiles are comparable, though the peak-to-peak value is higher in the measurement (34 mT) than in the simulation (18 mT; Fig. 2a; Supplementary Fig. 2d). This difference may be attributed to a number of factors, including the non-cubic shape of the real micro-magnets, an error in the estimate of the sample-to-probe distance, and a contribution from the side-wall deposits, neglected in the simulations. Nevertheless, the agreement is considered sufficient to support force simulations shown in Fig. 2a; Supplementary Fig. 2e,g, detailed below.

### Quantification of magnetic forces

First, evaluation of forces produced on one UML. The energy of interaction of the UML, with an induced magnetic moment **m**_UML_, and the total magnetic field, **B**_tot_, is: *E=−***m**_UML_·**B**_tot_ (ref. 35). The magnetic force **F***=−*∇ (*E*) is thus: **F**=∇ (**m**_UML_·**B**_tot_). The magnetic moments of the superparamagnetic nanoparticles in the UML are aligned along **B**_tot_ so: **F**=∇ (*m*_UML_*B*_tot_), where *m*_UML_ and *B*_tot_ are now scalar. UMLs at *z*=5±2 μm above the micro-magnet experience *B*_tot_ of ∼140 mT (the value is reduced compared to the external field value, because of the opposing induced field) and thus they are almost saturated (see Supplementary Fig. 2c and the magnetization curve of the UMLs in ref. 34). Thus, *m*_UML_ can be considered as almost constant, so that the force applied to a UML is proportional to the gradient of the amplitude of the magnetic field, and is given by: **F***=m*_UML_ ∇ (*B*_tot_). Note that because of the 8±4 × 10^−3^ concentration of UMLs found in cells after injection (see Evaluation of the number of UML inside mesoderm cells paragraph below), the mean distance between UMLs within cells is thus of the order of 10 times larger than their size (200 nm), so that attractive interactions between magnetized UML are very small compared with the force of interaction between the UMLs and the micro-magnets, given the (*d*/*r*)^3^ decay of the interactions between magnetic moments of magnetic systems of size *d* at a distance *r* (ref. 35). What is more, UMLs cannot aggregate, since they are trapped within the mesh of the cytoskeleton which is generally of the order of 70 nm (ref. 36). Effectively, no apical concentration of UMLs is specifically observed under the magnets after the application of the magnetic field pulses, in the *sna twi*-defective mutants in which the observation is made easier by the fact that invagination cannot be activated, demonstrating that the UMLs are trapped in the cytoskeleton (Supplementary Fig. 6b, *t*=435 s). In addition, as confirmed by Supplementary Fig. 1b, UMLs are inside cells, and the UMLs that would remain present out of the cell but within the embryo into the yolk will not significantly contribute to the force applied to the cells, because they are positioned at >20 μm from the magnet, that is, out of the specific range of the application of the force (magnetic forces decay strongly with the distance to the magnet, and practically, only UMLs in the 5 μm most apical part of the cell feel significant force, see the first section in the Methods section ‘Quantification of magnetic forces' and paragraph below with associated Supplementary Fig. 2f). In addition, there is no extracellular matrix out of the cells into the yolk, and more generally at this stage of development, to trap the UMLs. The magnetic force acting on the cytoskeleton is thus dominated by constricting/dilating forces produced by the UML–micro-magnet interactions, transmitted by the cytoskeleton in which UMLs are trapped, to the overall cell.

To evaluate the force applied on one UML, let us first consider the saturation values of magnetization and moment. The saturation magnetization of the constituent phase of the MNPs, maghemite, is *M*=3 × 10^5^ Am=0.38 T. Thus, the saturated moment of a 9 nm particle is *MV*=1.15 × 10^−19^ Am^2^. Based on magnetic measurements reported in the paper by Bealle *et al*., the UMLs, of average diameter 200 nm, are filled to roughly 20 vol.% with 9 nm diameter MNP. The saturation magnetization of a given UML is thus 76 mT, and its saturated moment 2.5 × 10^−16^ Am^2^. Calculations show that the magnetic field strength at 5 μm above the micro-magnet array is ∼0.14 T. From the *M(H)* curves of UML (Supplementary Materials section of ref. 34), 0.14 T gives 90% of saturation. From a theoretical point of view, a superparamagnetic spherical MNP has susceptibility limited either by its shape or by its Langevin susceptibility. The spherical MNP used here have a saturation magnetization of 0.38 T and a demagnetizing factor of 1/3, so the demagnetizing field is 0.38/3=0.12 T. If an UML is considered as a homogeneous magnetic sphere with magnetization 20% that of a MNP (since the UML is 20% full of MNPs), then its saturation magnetization is 20% of 0.38 T=76 mT. Thus, the demagnetizing field of an UML is 25 mT. The magnetic moment of a superparamagnetic particle is given by the Langevin function: *m*=*m*_s_*L*(*x*) with *x*=*m*_s_*μ*_0_*H*_appl_/*kT*. Using *m*_s_=1.15 × 10^−19^ Am^2^, *μ*_0_*H*_appl_=0.18 T and *T*=300 K, *x*=5 so *m*=80% *m*_s_. Thus, we consider that the UMLs and the MNPs are 80% saturated. *m*_UML_=2 × 10^−16^ Am^2^ is used in the force calculations. The force on a UML was calculated as a function of its position with respect to the micro-magnets using analytical expressions **F**=*m*_UML_ ∇ (*B*_*tot*_) (Matlab; Fig. 2a).

Second, measurement and simulation of force on a test object are described. To validate our force simulations, and to demonstrate the existence of repulsive forces in certain regions, we carried out force measurements on a test object scanned above the array of micro-magnets. The test object was a 1.5 μm sphere of polystyrene containing 30 volume % superparamagnetic nanoparticles, supplied by microParticles GmbH. The sphere was stuck to the apex of a commercial atomic force microscope cantilever using a micro-manipulator in a scanning electron microscope. The membrane containing the micro-magnet array was placed on the bulk magnet used for SHPM, so as to apply an in-plane field perpendicular to the axis of the linear array of magnets (that is, along y). The superparamagnetic microsphere was then scanned at different heights above the micro-magnet array, and the deflection of the probe along the *z* axis was measured as a function of position in the *x*–*y* plane (note that the probe was not operated in resonant mode). The level of deflection is directly proportional to the *z* component of the force exerted by the micro-magnets on the microsphere. As can be seen in Supplementary Fig. 2g, the sign of the force changes from attractive to repulsive, depending on the position of the sphere. The sphere is repelled when it is above the micro-magnets but attracted when displaced along the *y* axis, in the direction of the applied field. The force on the test object was calculated using analytical expressions (Matlab). Relatively good agreement is found between the measured and calculated forces. Good agreement was also found between the measured and calculated *z* derivative of the peak-to-peak force, as a function of *z* (Supplementary Fig. 2g). Though the approach used only allows us to measure the *z* component of the force, not the *y* component, it nevertheless validates our approach to simulations and demonstrates that both attractive and repulsive forces can be experienced in the geometry used in our bio-experiments, that is, with an in-plane field serving to magnetize the micro-magnets and the UML within the cells.

To quantify the number of UMLs, we measured the intensity of UML fluorescence per cell, subtracted from the background intensity away from the embryo. This led to local patterns of maximum intensity. Taking the smallest intensity maximum as a single UML signal, we evaluated 18±7 UMLs per cell in an optical section 0.5 μm-thick two-dimensional disc (measured in *N*=6 embryos, 494 cells, error bars being s.d.), namely, 540±210 UMLs in a 15 μm-thick cell of 5 μm apical diameter, or an UML concentration of 8±4 × 10^−3^.

Third, evaluation of forces produced on one cell is described. The force applied apically to UMLs in one cell to compress its neighbouring cell apex by 20% was thus estimated to be of the order of 0.5±0.2 nN (considering that 1/3 of the individual UML located in the 5 μm most apical part of the cell are submitted to a mean constricting force of 2 × 10^−12^ N (Fig. 2a)). This order of magnitude is in line with compression forces of 0.6 nN per cell (60 nN involved in the compression of 20% of nearly 100 full cells)^65^.

Note that the volume of the 3 μm diameter nucleus approximated as a sphere, that apically excludes UMLs, was evaluated to be ∼9 μm^3^, which remains small compared with the volume of the apical cylinder in which the nucleus is located (diameter=5 μm; length=5 μm so volume=98 μm^3^), and was thus not taken into consideration in the evaluation.

Consistently, applying a pulsating homogeneous magnetic field either in the absence of the micro-magnet array (Supplementary Fig. 5) or without injecting UMLs, resulted in neither pulsations nor medio-apical accumulation of Myo-II nor apical constriction. This is in agreement with the fact that the UML feel only a field gradient force, and that the micro-magnets are required to locally concentrate the magnetic field so as to produce localized field gradients. Magnetic field gradients also *a priori* apply a force normal to the tissue, which could is some cases be detected as leading to a maximum of 0.5 μm deformation. However, the mechanical induction of the medio-apical accumulation of Myo-II by normal deformation indentation requires a much higher perpendicular deformation of the tissue, of a minimum of 5 μm (ref. 18), ruling out any effect of the normal forces developed by the micro-magnets in the activation of the medio-apical accumulation of Myo-II.

### Imaging

A spinning disc (Yokogawa) microscope (IX70 Olympus) coupled to a CoolSnap HQ^2^ camera was used in all experiments using the 405, 491 and 561 nm length waves, as well as transmission. All settings, including the piezo-electric control of *z* position for stack production (Princeton Instrument), were controlled by the Metamorph software, all assembled by Roper. Images have been taken with a 40 times water objective (Olympus LUMPLFN40XW) and a 60 times oil objective (Olympus PLAPON60XO1.42). A 20 times air objective (Olympus UIPLSAPO20XO0.85) was also used for the injections and for some other observations.

Note that the 50 μm-thick PDMS membrane containing the micro-magnets introduces a new optical index medium in between the glass coverslip and the halocarbon oil in which the embryos are immerged, and that diffraction is introduced by the presence of the 10 μm iron cubes every 20 μm. This introduces noise in the images that however does not prevent cell resolution analysis. Note also that the embryos are not flattened against the coverslip or PDMS to avoid any mechanically inducted interference due to the squeezing. Thus invaginations generated large movements that frequently obliged the operator to re-centre the image to not lose the cell apexes. This prevented also the use of × 60 magnification during the constriction phase, to not lose the imaging field and mean focalization plan. The use of × 40 introduced pixelarization in some cases.

### Image analysis

Apical cell shapes were imaged at junctions, 3 μm in *z* (in depth) below the apical surface of the epithelial cells. Individual apical cell areas were measured using FIJI and Freed software. Time lapse was realized *in vivo* every 3 s using spinning disc imaging of the apical domain of the cells of the ventral side of *Drosophila* embryos labelled with *spider–GFP* and *sqh-Cherry*. Individual cell tracking were realized afterwards, *in silico*. Individual cells were manually tracked. The observation started 120 s before the end of ventral cellularization, and ended 300 s after, at the beginning of germ-band extension. Matlab software was used to execute Fourier transform on the dynamics of individual apex cell shape changes. No filtration was applied to generate the Fourier transform. The drift (mean slope of the basal corresponding to stable constriction that adds to pulsations) was removed, to analyse the signal that is associated to pulses only. Mean values and graphs were realized with Kaleidagraph software.

Time-lapse resolution is of the order of 1 min for whole mesoderm imaging. 

Fluorescent intensity was quantified by subtracting the background intensity (away from the embryo) from the signal measured in cells. Correlation functions between distant pixels were evaluated using FIJI ( http://imagejdocu.tudor.lu/doku.php?id=macro:radially_averaged_autocorrelation; http://rsb.info.nih.gov/ij/plugins/radial-profile.html). Author Paul Baggethun.

Kymographs were realized in transmission, using ImageJ.

### *Drosophila* mechanical indents

A micromanipulated 50 μm diameter needle was approached close to the mesoderm of stage 5. In the specific case of *sna-/Cyo;sqh-GFP* embryos. Embryos having not initiated apical accumulation of Myo-II 2 min after the end of ventral cellularization were genotyped as *sna* homozygous. Embryos were systematically indented by 3–7 μm during 4 min at the most posterior 1/3 position of the mesoderm, 2-3 min after the end of cellularisation. After stimulation, the embryo was turned 90° for observation of the ventral side.

### Statistics

All statistics based on quantitative measurements were evaluated with the non-parametric two-sided Mann–Whitney test, with s.d. error bars being comparable within each group of data, and the variance between groups of data being similar. Error bars are s.d., except in Fig. 3f in which they are s.e. Most of the experiments were reproduced at least six times in the laboratory, which is standard in single embryo *in vivo* experiments. *n* is the number of measurements per experiment. All statistics based on qualitative measurements (on/off results, that is, the presence of an invagination or not) were evaluated with the exact Fisher test.

### Data availability

The authors declare that all data supporting the findings of this study are available within the article and its Supplementary Information files or from the corresponding author upon reasonable request.

## Additional information

**How to cite this article:** Mitrossilis, D. *et al*. Mechanotransductive cascade of Myo-II-dependent mesoderm and endoderm invaginations in embryo gastrulation. *Nat. Commun.* **8,** 13883 doi: 10.1038/ncomms13883 (2017).

**Publisher's note:** Springer Nature remains neutral with regard to jurisdictional claims in published maps and institutional affiliations.

## Supplementary Material

Supplementary Information Supplementary Figures and Supplementary Reference

Supplementary Movie 1 WT, Spider-GFP Myo-II-mCherry, stage 6, partial-zoom (apical resolution + collective effect), movie duration 606s.

Supplementary Movie 2 Sna Twi Rnai, Spider-GFP Mtyo-II-mCherry, stage 6, un-zoomed (no constricting cell) movie duration 900s.

Supplementary Movie 3 Sna Twi Rnai, Spider-GFP, UML-Rhodamin, stimulated magnetically, zoomed (pulsation of cells neighbouring the micro-magnets), movie duration 669s.

Supplementary Movie 4 WT, Spider-GFP, Myo-II-mCherry, movie duration 606s.

Supplementary Movie 5 Sna Rnai, Spider-GFP, Myo-II-mCherry, movie duration 534s.

Supplementary Movie 6 Sna Rnai, Spider-GFP, Myo-II-mCherry, stimulated magnetically, movie duration 720s.

Supplementary Movie 7 Sna Twi Rnai, Spider-GFP, Myo-II-mCherry, stimulated magnetically, movie duration 900s.

Supplementary Movie 8 Sna Twi Rnai, Spider-GFP, Myo-II-mCherry, UML blue, stimulated magnetically, movie duration 900s. The discontinuities in the movies have the same origins as in Movie 9a,b.

Supplementary Movie 9 WT, Spider-GFP, movie duration 780s.

Supplementary Movie 10 Sna Rnai, Spider-GFP, movie duration 774s.

Supplementary Movie 11 Sna Rnai, stimulated magnetically, Spider-GFP, UML-Rhodamin, movie duration 678s.

Supplementary Movie 12 Sna mutant, Myo-II-GFP, Indent, movie duration 576s.

Supplementary Movie 13 Sna Fog Rnai, Myo-II-GFP, stimulated magnetically, movie duration 1068s.

Supplementary Movie 14 WT, Rok-GFP, movie duration 1400s.

Supplementary Movie 15 Sna Rnai, Rok-GFP, movie duration 1400s.

Supplementary Movie 16 Sna Rnai, Rok-GFP, stimulated mechanically, movie duration 1380s.

Supplementary Movie 17 Sna Fog Rnai, Rok-GFP, stimulated mechanically, movie duration 1080s.

Supplementary Movie 18 Time-lapse gastrulation phenotype of a wild-type in transmission light, movie duration 8910 seconds.

Supplementary Movie 19 Time-lapse gastrulation phenotype of a bicoid nanos torso RNAi injected embryo in transmission light, movie duration 8910 seconds.
