# Supplementary material for: Mechanotransductive cascade of Myo-II-dependent mesoderm and endoderm invaginations in embryo gastrulation
Source: Nat Commun. 2017 Jan 23;8:13883. doi: 10.1038/ncomms13883 (PMC5264015; doi:10.1038/ncomms13883)
Supplement: Supplementary Information — Supplementary Figures and Supplementary Reference [file ncomms13883-s1.pdf]

Supplementary Figures

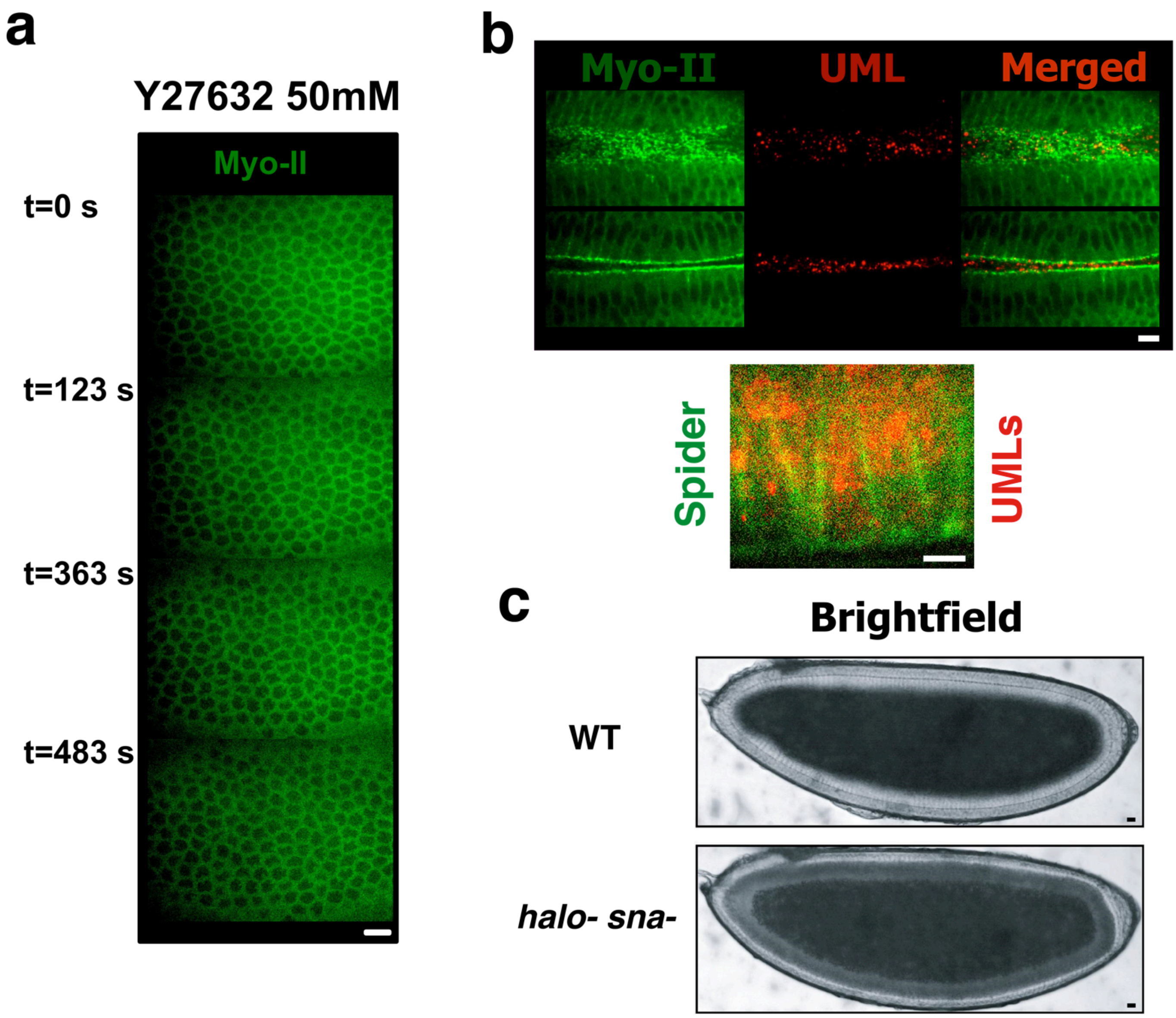

Supplementary figure 1: Myo-II dependence of sna-dependent mechanical pulsations, magnetic loading for mimicking mechanical pulses and *halo sna* mutant phenotypes. a Inhibition of *sna*-dependent pulses in embryos injected with the Y27632 inhibitor of Rock and of Myo-II activity. Characteristic of n=10 on N=12 tested embryos ($p<10^{-3}$, exact Fisher test given the fact that all WT show apical stabilisation of Myo-II and mesoderm invagination). **b** Myo-II-GFP WT embryos injected with Rhodamin labelled UMLs: **Up** ventral view at gatstrulation. Scale bar is 10µm **Down:** Zoomed lateral view, showing UMLs inserted into the cells at cellularization completion (the full cell length is of 15 microns at this stage). Scale bar is 5µm. **c** Genotyping *a posteriori* homozygous *sna* mutant embryos before manipulation by using halo phenotypes. The *halo-* mutation shows, when homozygous, a defect of transport of lipid droplets out of the cells during cellularisation[1]. The associated phenotype consists in a grey halo of droplets between cell apexes and basal membrane, compared to the WT, which is observed during cellularisation, before gastrulation. Following reference[1], *sna* homozygous mutants were selected from the progeny of double mutation on the same chromosome *halo sna /Cyo;sqh-GFP* progenitors, in genotyping them by using the phenotype showing invasion of lipid droplets inside cellularizing cells (cell base in grey) at stage 5, compared to the WT phenotype in which cells are depleted of lipid droplets. Scale bar is 10µm.

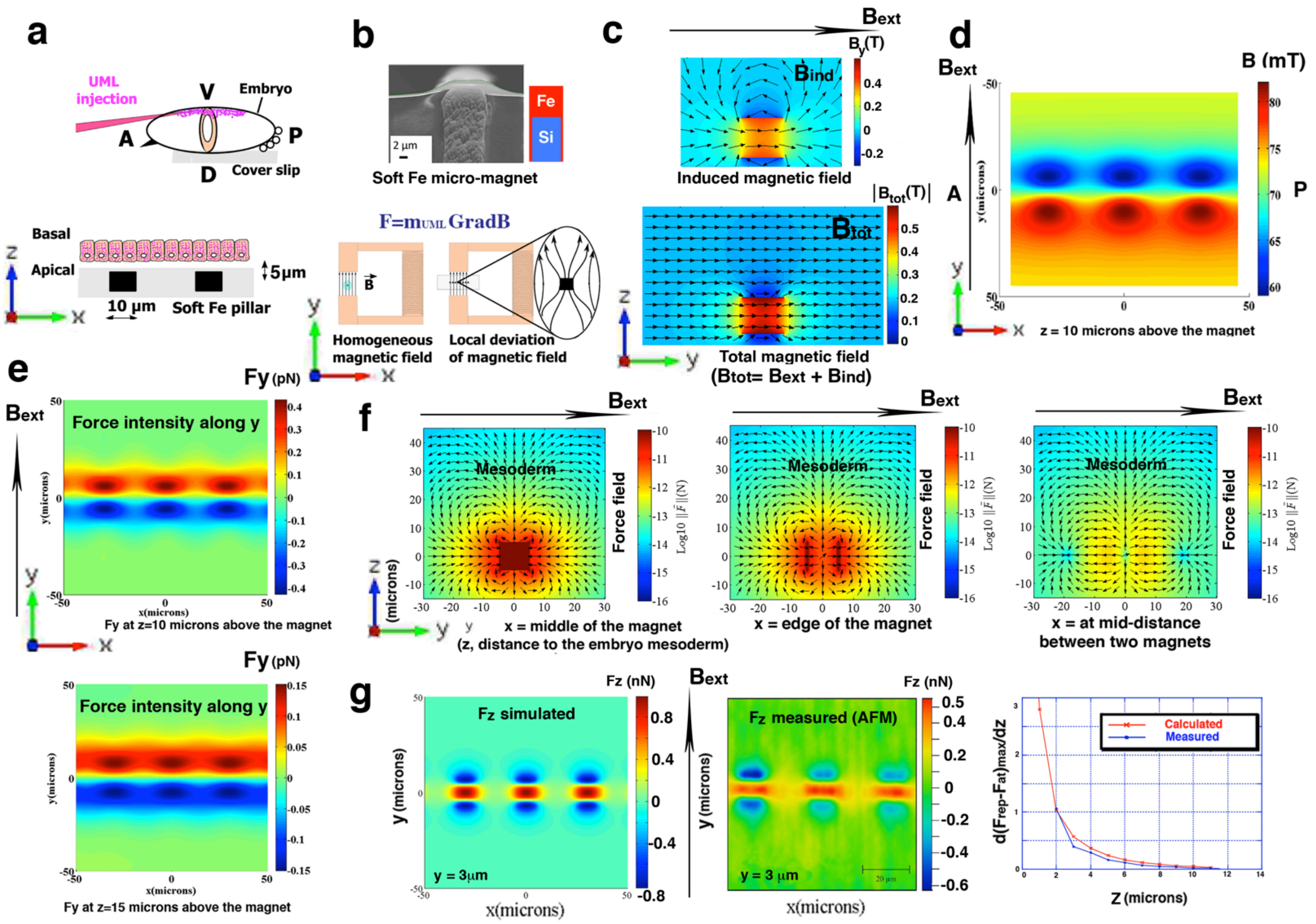

Supplementary figure 2: Magnetic field produced in the vicinity of an array of micro-magnets, and the resulting field gradient forces produced by the micro-magnets on an UML, as a function of the position of the UML with respect to the micro-magnets. Intensity is given by colour code, the direction of the field or force is given by arrows of normalised length. The micro-magnets are magnetised by an external magnetic field applied in the plane of the micro-magnet array perpendicular to the array axis. **a Up:** schematic of UML injection along the basal side of mesoderm epithelial cells, open to the yolk at stage 5, **down:** and of the relative disposition of UML loaded mesoderm epithelial cells with respect to the linear array. **b Up**: Electron microscope image of an individual soft iron micro-magnet and schematic of a cross-sectional cut. **Down:** schematic of how the soft iron micro-magnets locally concentrate the magnetic field lines (perpendicular to the objective in blue) produced by an electro-magnet, leading to the magnetic field gradients (**Grad***B*). These field gradients generate magnetic forces **F** on the UML here shown in the (x,y) plane crossing the iron pillar. **c Up :** stray field B_{ind} from micro-magnet. **Down:** total magnetic field (externally applied B_{ext} + micro-magnet B_{ind}). **d** Simulation of the stray field values corresponding to Fig. 2a configuration, produced at a height of 10 µm above an array of 5 micro-magnets, on the apical surface of mesoderm cells. A is anterior and P is posterior. **e** Y component of the force on one UML in the horizontal plane (x-y plane) positioned 10 µm and 15 µm above the plane of the array (at z = 10 and 15 microns, respectively), on the apical surface of mesoderm cells. **f** Force in the y-z plane, at positions x=0 microns (midpoint of the central cube), at position x=5 microns (edge) and x=15 microns (mid point between the central cube and an edge cube). Forces are negligible along the x-axis. **g Left and centre:** Simulation and MFM measurement of the z component of F applied on a 1.5µm bead (30% iron oxide), in the x-y plane at z = 3 microns; **Right:** quantitative comparison between measurement and calculation of the maximum repulsive force minus the maximum attractive force on the UML in a given x-y plane (derivative is used to remove background effects).

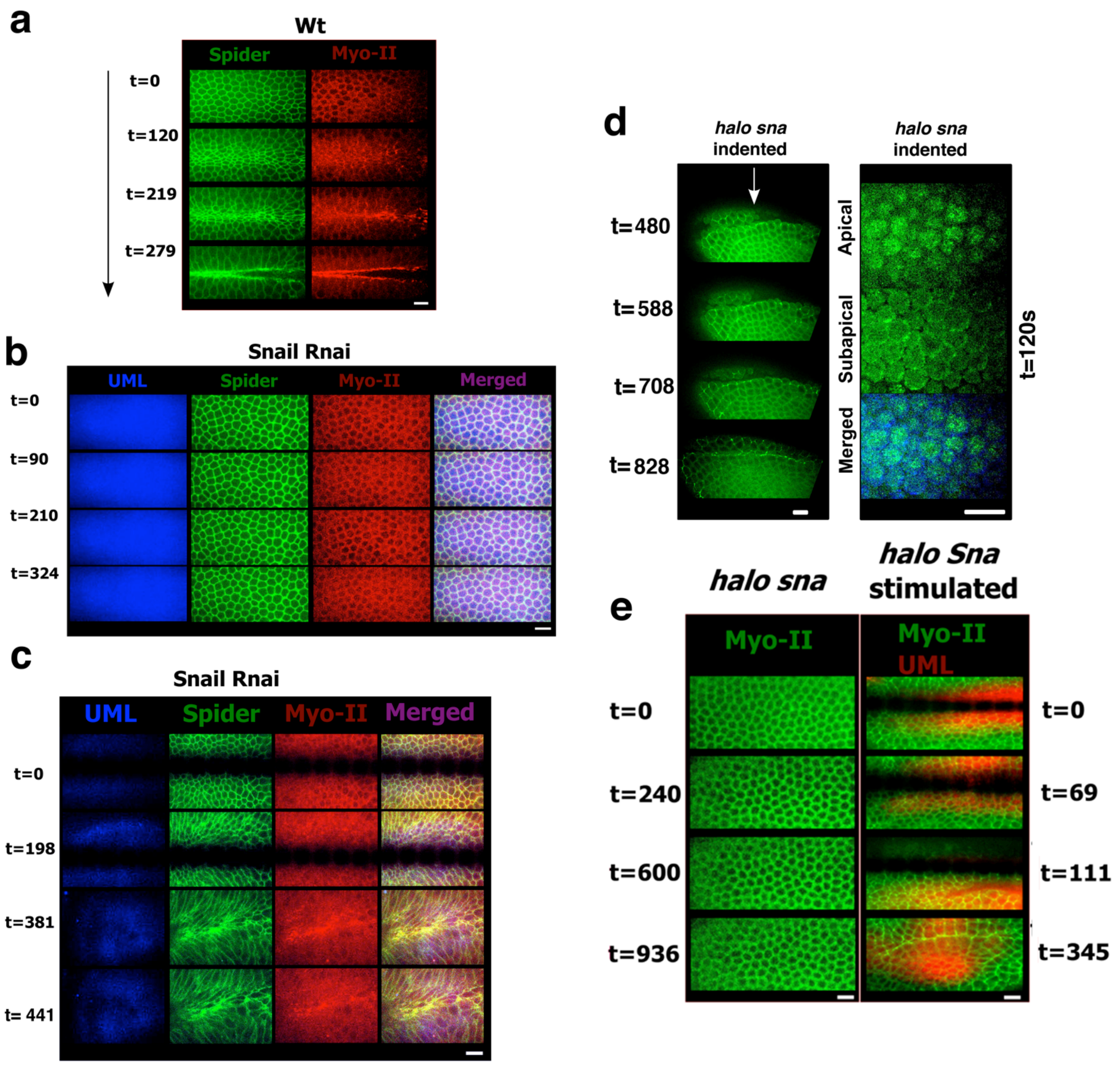

Supplementary figure 3: Rescuing medio-apical accumulation of Myo-II, coordinated apical constriction and mesoderm invagination in *sna* defective embryos by magnetically stimulated mimicking of *sna* dependent pulsations, in Sider-GFP Myo-II-mCherry embryos. a Coordinated apical constrictions (Spider, Gilgamesh in green) and Myo-II stabilisation (in red) followed by mesoderm invagination in the WT (representative of all embryos), **b** all effects observed in part "a" are absent in *sna* RNAi injected embryos injected with blue labelled UML, representative of n=6 of the N= 6 embryos observed, **c** and are rescued by magnetically stimulated mimicking of *sna* dependent pulsations, representative of n=3 of the N= 5 embryos observed (p= 5.10^{-3} exact Fisher test given N=12 *sna* RNAi injected embryos showing no apical stabilisation of Myo-II of Figs. 1e and 4a, full invagination (n= 1), partial invagination (n= 2)). **d** Mechanical rescue of apical stabilisation of Myo-II and mesoderm folding in *halo sna* mutant embryos by local indent: *halo sna- Myo-II-GFP* embryo indented 3 minutes after the end of ventral cellularization completion, and four minutes before the movie initiation and rotated 90°. Representative of n=4 of the N=10 embryos observed (p=3.10^{-2}, exact Fisher test given the n=11 non invaginated on the N=11 *halo sna* control observed – see below)). The invagination is shown by a white arrow. Observations are realized with a *60 objective. Zoom on the mesoderm of the indented *halo sna-Myo-II-GFP* embryo. Representative of n=7 of the N=10 embryos observed (p<10^{-3}, exact Fisher test). **e** Apical accumulation of Myo-II and mesoderm invagination after the initiation of magnetic stimulation of *sna*-dependent pulsations in *halo sna* embryos (Myo-II in green, UML in red). Representative of n=16 of the N=20 *halo snail* embryos (Myo-II apical accumulation (n=16), full invagination (n=8), partial invagination (n=8)), compared to the *halo sna Myo-II-GFP* embryos non-invaginating (n=11 on the N=11 observed). p<10^{-3}, exact Fisher test. Scale bar is 10µm.

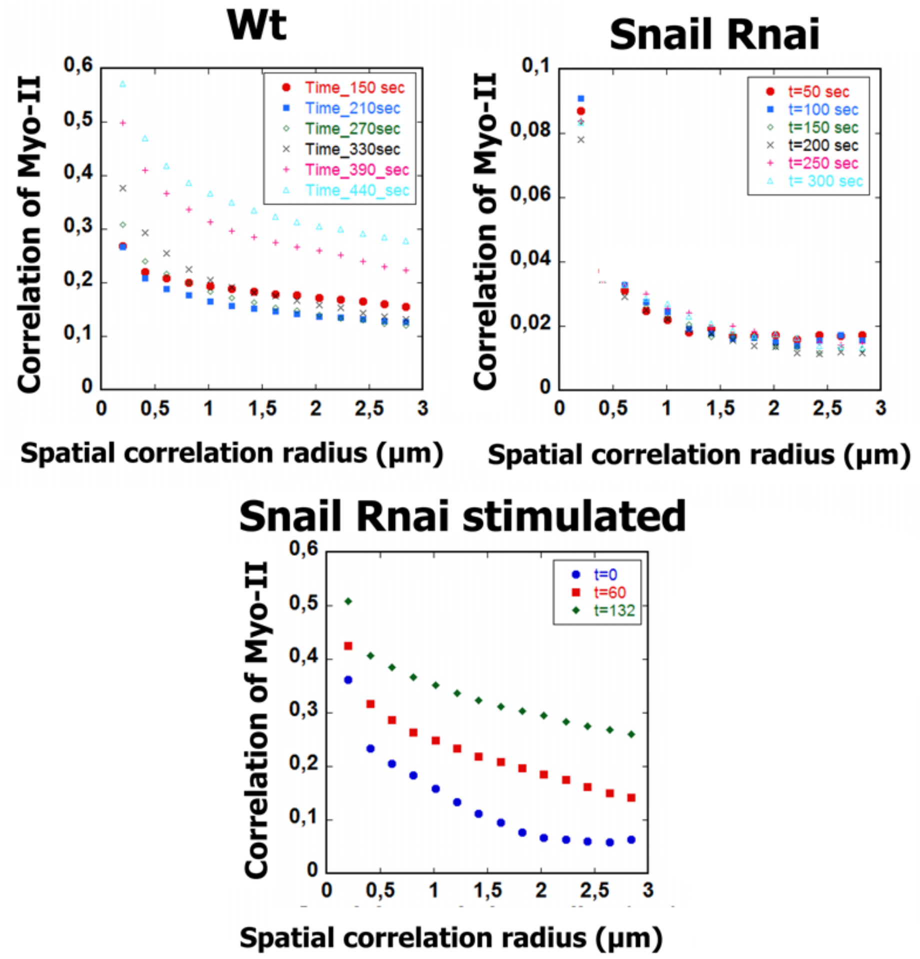

Supplementary figure 4: Temporal evolution of the correlation of apical constriction as a function of the distance inside cells during the first phase of mesoderm invagination. The spatial correlation of Myo-II spots is the probability to find a spot of Myo-II at a distance *r* from a given spot into the surface of the apexes. The increase of correlation with the decrease of the distance *r* shows that the probability of production of a spot is increased due to the presence of another spot located at the distance *r* in the WT, *i.e* a process of correlative auto-inductive production of spots. The spatial coordination of apical accumulation of Myo-II inside mesodermal cells in the WT increased by almost 100%, 440 s after the initiation of pulsations, beginning at 330 s, *i.e.* at the beginning of the coordinated stable constriction wave, showing the increase of the correlative auto-inductive production of spots (Fig. 1b). The correlation decreased at short time scale, and no correlation increase was found in *sna* RNAi injected embryos, showing no increase of correlative auto-inductive production of spots, consistent with a lack of mechanical stabilisation of Myo-II in the absence of snail dependent pulsations. The correlation at short time scale, and the correlation increase of nearly 100% was rescued in cells located within 20 µm of the micro-magnets, 130 s after magnetic stimulation in *sna* RNAi injected embryos, showing the mechanical rescue of the correlative auto-inductive production of spots. This confirms that *snail*-dependent pulsatile mechanical strains due to medio-apical pulsatile spots of Myo-II initiate the mechanotransductive stabilisation of medio-apical stabilisation of Myo-II, with stabilized spots amplifying in turn the process. Representative of N=8 WT embryos, N=12 *sna* RNAi injected embryos and N=3 *sna* RNAi plus N=2 *halo sna* embryos magnetically stimulated.

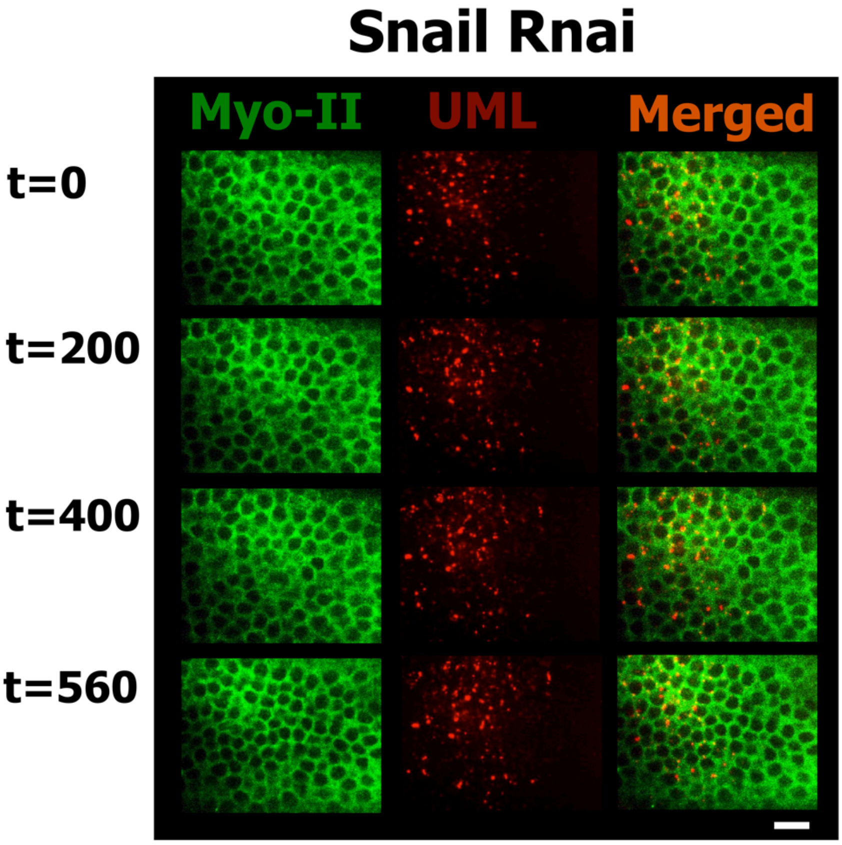

Supplementary figure 5: Application of a pulsating magnetic field in the absence of the micro-magnet array does not induce apex deformation nor invagination rescue in *sna* defective embryos. Myo-GFP embryos injected with Sna Rnai and UML and submitted to a pulsating magnetic field, spatially homogeneous at the scale of the embryo. The field is “on” (180 mT) from t=40 s to t=120 s and from t=160 s to t=240 s (“off” = 0 T). Scale bar is 10µm.

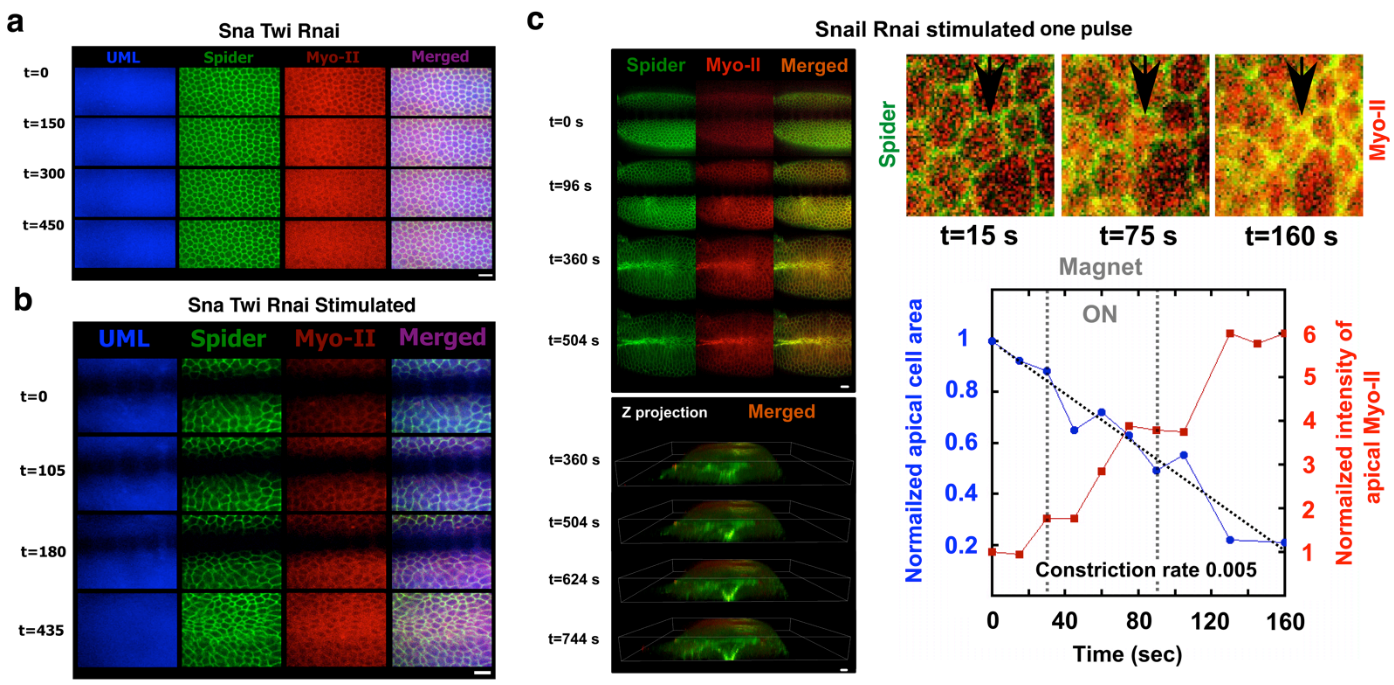

Supplementary figure 6: Mechanical stimulation of: *sna twi* defective embryos; *sna* defective embryos with a single pulse. a,b Magnetically stimulated mimicking of *sna* dependent pulsations in *sna twi* defective embryos induces cell deformation with no apical accumulation of Myo-II and no invagination rescue. **a** Spider GFP Myo-II-mCherry embryos injected with *sna* RNAi, *twi* RNAi and blue labelled UML. Representative of n= 6 of the N= 6 embryos observed. **b** Spider GFP Myo-II-mCherry embryos injected with *sna* RNAi, *twi* RNAi and blue labelled UML with magnetically stimulated rescue of *sna* dependent pulses. Representative of n=2 of the N= 2 embryos observed (p=0.04, exact Fisher test). **c** Magnetically stimulated mimicking one *sna* dependent pulse in *sna* defective embryos is sufficient to rescue mesoderm invagination. The 60s single pulse was applied at 36s. Representative of n=6 embryos on N=9 embryos tested ($p<10^{-3}$, exact Fisher test, given N=18 *sna* RNAi injected embryos showing no apical stabilisation of Myo-II of Figs. 1e, 4a and Supplementary figure 3b). Zoom: Myo-II medio-apical stabilisation. Quantification: tracking on a representative cell. Scale bar is 10µm.

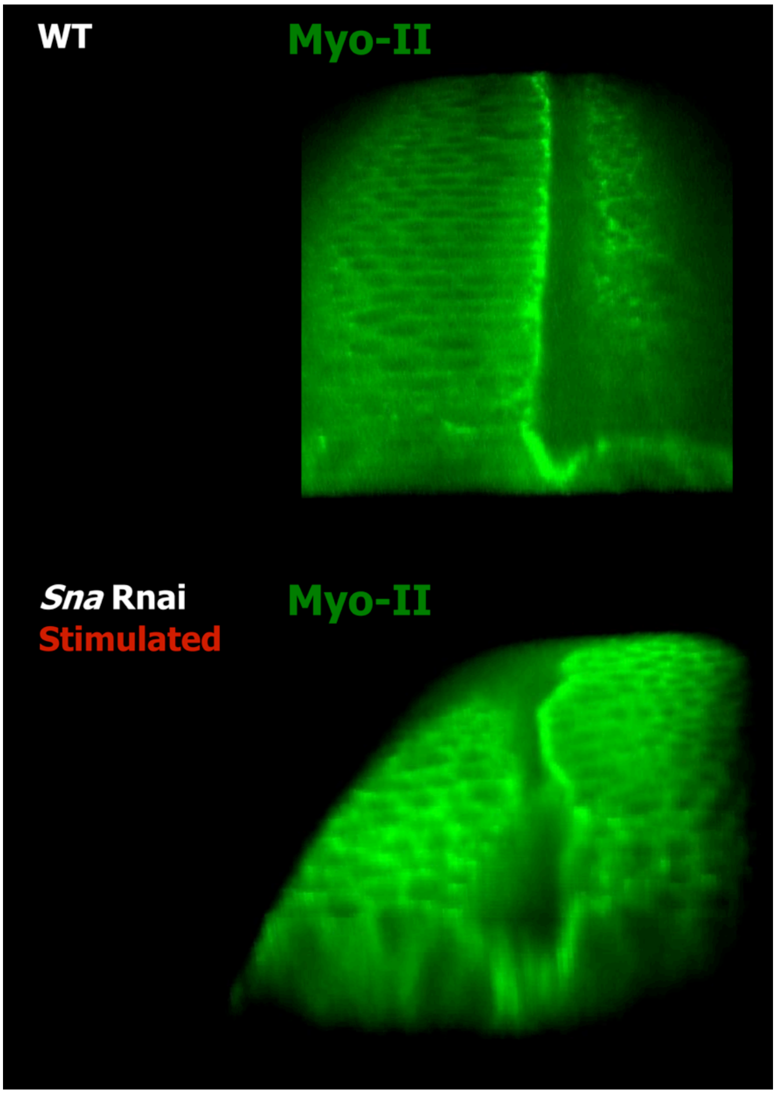

Supplementary figure 7: 3D reconstruction of mesoderm invagination initiation in the *sna* defective embryos mechanically stimulated in comparison to the WT. Up : WT. Down : sna Rnai. Myo-II-GFP is shown in green.

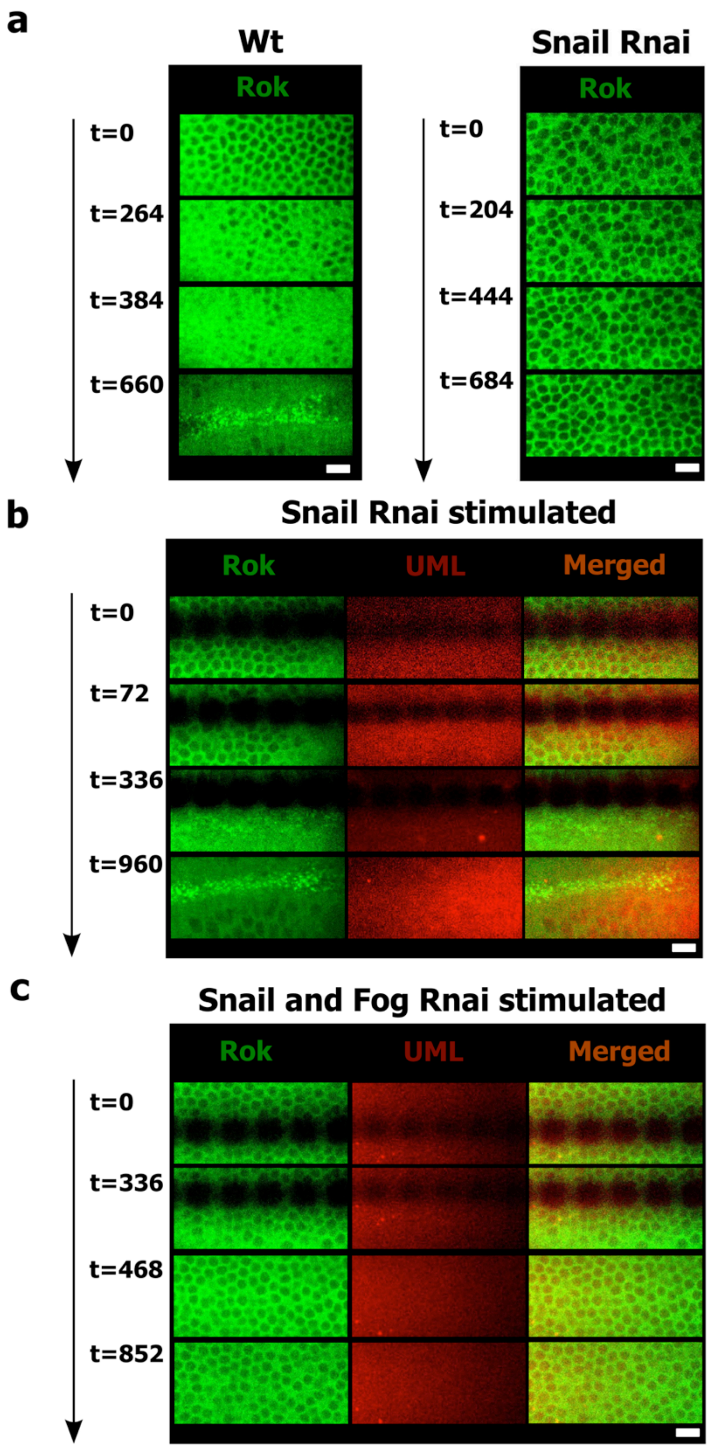

Supplementary figure 8: Fog dependent medio-apical accumulation of Rok rescue, by magnetically stimulated mimicking of *sna* dependent pulsations in *sna* defective embryos. a Rok-GFP embryos, without (N=6) and with RNAi injection (representative of n=6 of the N=7 embryos, p=4.10^{-3}, exact Fisher test). **b** UML loaded Sna RNAi Rok-GFP embryos magnetically stimulated as in part Fig.5b. Representative of n=9 of the N=14 embryos (p=0.01)**. c** UML loaded Sna RNAi Fog RNAi Rok-GFP embryos magnetically stimulated as in Fig.5c. Representative of n=7 of the N=8 embryos (p=0.03). The micro-magnet array was removed after 336 s, so as to image the mesoderm directly above the individual micro-magnets. Scale bars are 10µm.

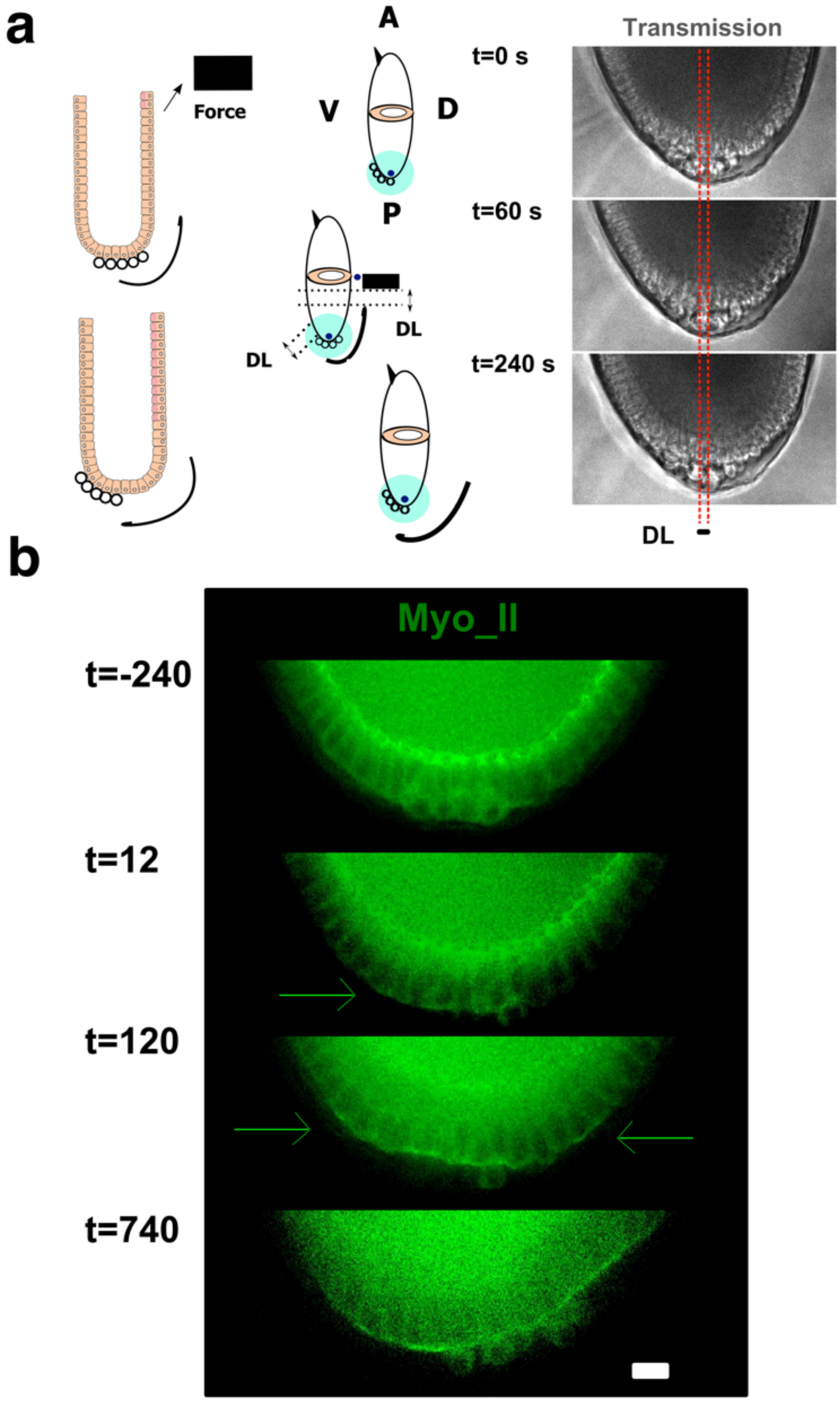

Supplementary figure 9: Myo-II apical stabilisation by stretching from the dorsal side of the embryo. a Mechanical rescue of posterior endoderm stretching by highly localised magnetic interactions with UML loaded in the dorsal epithelium of *halo sna* mutants. **b** Distribution of Myo-II-GFP in sqh-GFP, *halo sna* sqh-GFP magnetically stretched posterior endoderm by magnetic forces applied from the dorsal side of the embryo. Representative of the n=7 *halo sna* stretched embryos on N=11 tested (p=0.03, exact Fisher test given *halo sna* n=6 on N=6 of Fig.6e control). Scale bar is 5µm.

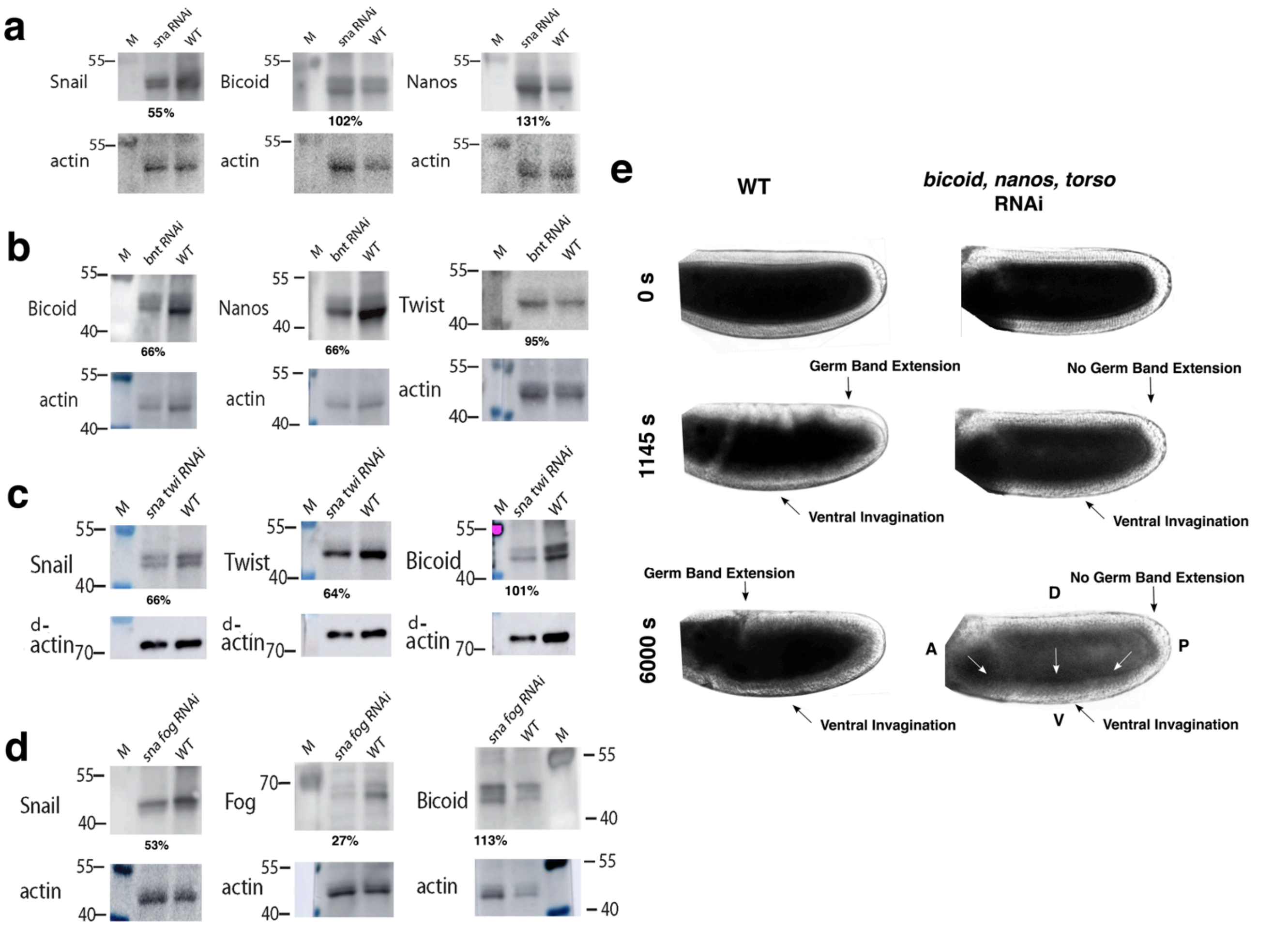

Supplementary figure 10: Quantification of mean protein level depletion during the overall 45min of stage 5, after RNAi injection at stage 4, for: **a** Sna in *sna Rnai* injected with Bicoid and Nanos as controls, **b** Bicoid and Nanos in *bicoid Rnai* and *nanos Rnai* injected with Twist as a control, **c** Sna and Twist in *sna Rnai* and *twist Rnai injected* with Bicoid as a control (here the nearly 50kD band did not reveal, possibly due to the stripping procedure: the so called d-actin more than 70kD band, probably showing actin dimers, or alternatively cross-reacting with another protein, was used as the loading control) **d** Sna and Fog in *sna Rnai* and *fog Rnai injected* with Bicoid as a control. Quantitative values are percentages normalized to Actin compared to the WT, after normalization to the actin loading control. The stripping procedure having allowed to label Actin with no overlapping labelling with the other target antibodies, sometimes introduced noise in the western, which did not prevent quantification. A slight overexpression of Nanos is observed in *sna* RNAi mutants, possibly due to the high noise on Actin in this specific case, but no repression. Note that weight markers can in a few cases be more visible on the uncropped whole westerns of Supplementary figure 11, on the right or on the left of the western. **e** Phenotype of the *bicoid Rnai*, *nanos Rnai, tor Rnai* embryos injected from the anterior, showing mesoderm invagination (white arrows and black arrow below) but no germ-band extension beginning of stage 7 (t=1145s) compared to the control, a phenotype stable a least until to end of stage 9 (6000s). Characteristic of all embryos (N=5, $p<10^{-3}$, exact Fisher test).

sna RNAi

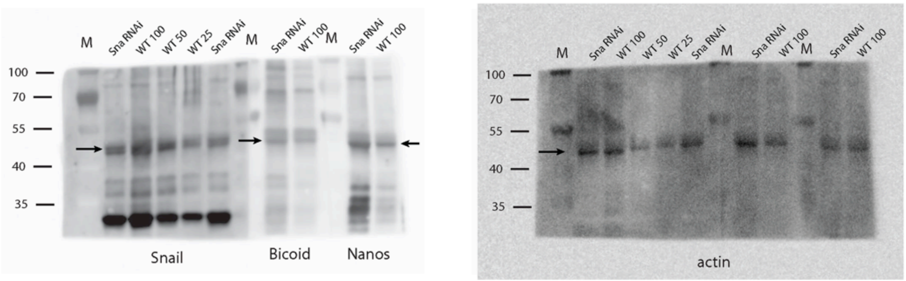

bnt RNAi

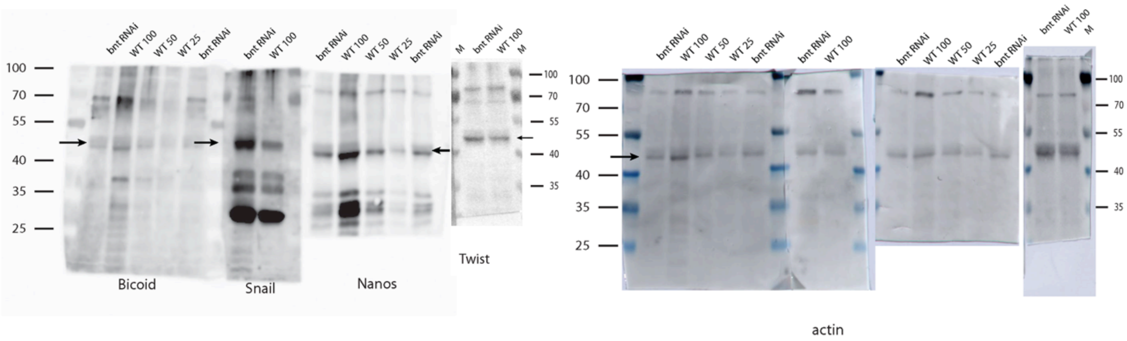

sna twi RNAi

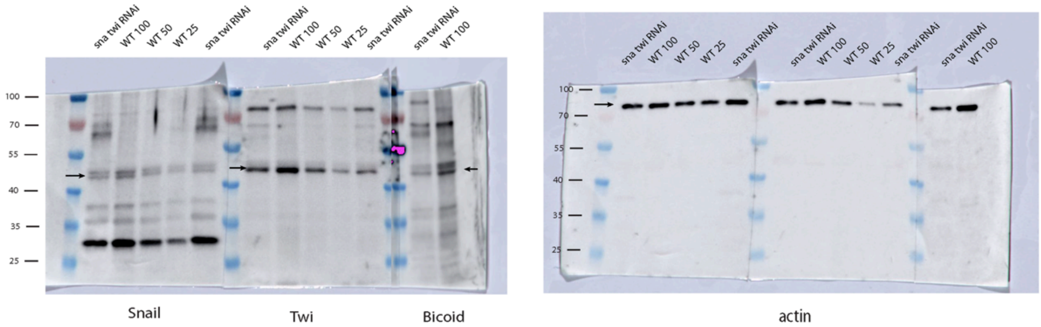

sna fog RNAi

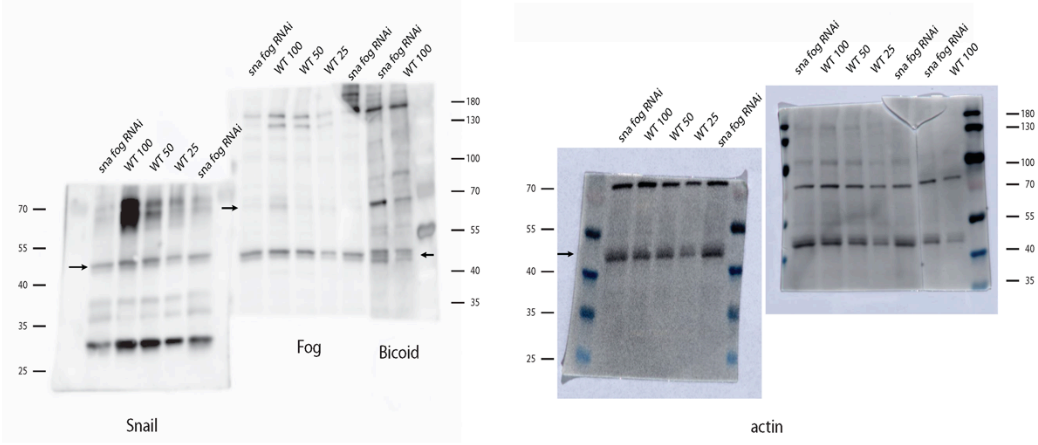

Supplementary figure 11: un-cropped raw western blots. Note that diluted concentrations of WT to 50% (WT50) and 25% (WT25) of the 100% (WT100) concentration were often used to validate for the sensitivity of quantitative evaluations to the concentration of the proteins at the conditions found.

Supplementary Reference:

1. Martin, A. C., Kaschube, M. & Wieschaus, E. F. Pulsed contractions of an actin-myosin network drive apical constriction. *Nature* **457**, 495-499 (2009).
